# Antibody Fc-binding profiles and ACE2 affinity to SARS-CoV-2 RBD variants

**DOI:** 10.1007/s00430-023-00773-w

**Published:** 2023-07-21

**Authors:** Ebene R. Haycroft, Samantha K. Davis, Pradhipa Ramanathan, Ester Lopez, Ruth A. Purcell, Li Lynn Tan, Phillip Pymm, Bruce D. Wines, P. Mark Hogarth, Adam K. Wheatley, Jennifer A. Juno, Samuel J. Redmond, Nicholas A. Gherardin, Dale I. Godfrey, Wai-Hong Tham, Kevin John Selva, Stephen J. Kent, Amy W. Chung

**Affiliations:** 1 Department of Microbiology and Immunology, The Peter Doherty Institute for Infection and Immunity, The University of Melbourne, Melbourne, VIC 3000 Australia; 2 The Walter and Eliza Hall Institute of Medical Research, Parkville, Melbourne, VIC Australia; 3 Immune Therapies Group, Burnet Institute, Melbourne, VIC Australia; 4 Department of Clinical Pathology, University of Melbourne, Melbourne, VIC Australia; 5 Department of Immunology and Pathology, Central Clinical School, Monash University, Melbourne, VIC Australia; 6 Department of Medical Biology, University of Melbourne, Melbourne, VIC Australia; 7 Melbourne Sexual Health Centre, Department of Infectious Diseases, Central Clinical School, Monash University, Melbourne, VIC Australia

**Keywords:** Receptor binding domain, SARS-CoV-2, Antibody, Variants, Omicron, IgG, Fcγ receptors, ACE-2, Affinity

## Abstract

Emerging SARS-CoV-2 variants, notably Omicron, continue to remain a formidable challenge to worldwide public health. The SARS-CoV-2 receptor-binding domain (RBD) is a hotspot for mutations, reflecting its critical role at the ACE2 interface during viral entry. Here, we comprehensively investigated the impact of RBD mutations, including 5 variants of concern (VOC) or interest—including Omicron (BA.2)—and 33 common point mutations, both on IgG recognition and ACE2-binding inhibition, as well as FcγRIIa- and FcγRIIIa-binding antibodies, in plasma from two-dose BNT162b2-vaccine recipients and mild-COVID-19 convalescent subjects obtained during the first wave using a custom-designed bead-based 39-plex array. IgG-recognition and FcγR-binding antibodies were decreased against the RBD of Beta and Omicron, as well as point mutation G446S, found in several Omicron sub-variants as compared to wild type. Notably, while there was a profound decrease in ACE2 inhibition against Omicron, FcγR-binding antibodies were less affected, suggesting that Fc functional antibody responses may be better retained against the RBD of Omicron in comparison to neutralization. Furthermore, while measurement of RBD–ACE2-binding affinity via biolayer interferometry showed that all VOC RBDs have enhanced affinity to human ACE2, we demonstrate that human ACE2 polymorphisms, E35K (rs1348114695) has reduced affinity to VOCs, while K26R (rs4646116) and S19P (rs73635825) have increased binding kinetics to the RBD of VOCs, potentially affecting virus–host interaction and, thereby, host susceptibility. Collectively, our findings provide in-depth coverage of the impact of RBD mutations on key facets of host–virus interactions.

**Supplementary Information:**

The online version contains supplementary material available at 10.1007/s00430-023-00773-w.

## Introduction

Severe acute respiratory syndrome coronavirus 2 (SARS-CoV-2)—the virus responsible for causing Coronavirus Disease 2019 (COVID-19)—remains an ongoing challenge for global public health, as genomic surveillance continues to identify new mutations within the SARS-CoV-2 genome. Mutations have previously given rise to new variant of concern (VOC) lineages, namely Alpha (B.1.1.7), Beta (B.1.351), Gamma (P.1), Delta (B.1.617.2), and now currently Omicron (B.1.1.529), each demonstrating increased transmissibility, resulting in new waves of infections across the world [1, 2]. Mutations to the receptor-binding domain (RBD) of the SARS-CoV-2 spike protein are found across all VOC lineages and are of great interest [1]. Given that binding of the SARS-CoV-2 RBD to angiotensin-converting enzyme 2 (ACE2) receptor expressed on host cells facilitates viral entry and subsequent infection, RBD mutations such as N501Y (found on several VOCS including Alpha, Beta, Gamma, and Omicron) can enhance the virus’ binding affinity to ACE2, and boost SARS-CoV-2 infection and transmission [3, 4].

Antibodies play crucial roles in the body’s immunological response against SARS-CoV-2 infection. Neutralizing antibodies block engagement to ACE2 receptor on host cells, thus protecting from symptomatic infection [5, 6]. Strong neutralizing activity against the ancestral SARS-CoV-2 RBD has been observed following seroconversion during SARS-CoV-2 infection, and COVID vaccination, particularly after receiving mRNA vaccines (such as Comirnaty BNT162b2) [5, 7]. However, it is well established that reduced neutralization capacity to VOCs, including Beta, Delta, and especially Omicron, are observed following both natural infection and vaccination [8–10]. Loss of epitope recognition by antibodies due to RBD mutations, such as the E484K (RBD mutation found in Beta and Gamma), consequently reduces neutralizing capacity by antibodies [11]. Omicron and its sub-variants harbor > 16 amino-acid changes at the RBD; and demonstrated substantial escape from existing neutralizing antibodies [12].

While RBD escape mutations can allow SARS-CoV-2 to evade neutralization, many of the antibodies elicited to the RBD are non-neutralizing [13, 14]. Antibodies can coordinate additional functions via the crystallizable fragment (Fc) region. Growing evidence supports roles for Fc-mediated immunity in control of SARS-CoV-2 infection [15–19]. Engagement of IgG with the IgG Fc gamma receptor (FcγR) on the surface of innate immune cells induces downstream effector functions, including antibody-dependent cellular phagocytosis (ADCP) (via FcγRIIa), and antibody-dependent cellular cytotoxicity (ADCC) (via FcγRIIIa). ADCP has been described to occur following infection and vaccination in humans to ancestral SARS-CoV-2 [14]. Critical importance for Fc-mediated immunity has been highlighted in animal studies, where animals that received neutralizing monoclonal antibodies (mAbs) with compromised Fc regions demonstrated greater viral burdens [15, 18, 20]. Furthermore, recent murine SARS-CoV-2 mRNA vaccine studies have demonstrated the essential role of FcR for protection against Omicron infection [21]. Despite the growing roles for Fc-mediated immunity in SARS-CoV-2, the impact of RBD mutations on the capacity for antibodies to recruit these functions is minimally described. Greater understanding of antibody Fc-effector functions to VOCs would allow insights into potential protective antibody signatures that remain following vaccination or infection across emerging viral variants.

Moreover, while mutations within the RBD can alter the virus’ affinity for ACE2, naturally occurring ACE-2 polymorphisms found within the human population, could also modulate host–pathogen dynamics during SARS-CoV-2 infection [22–25]. Numerous human ACE2 (hACE2) protein-altering single-nucleotide polymorphisms have previously been characterized with low frequency among the human population—including E35K (rs1348114695), K26R (rs4646116), and S19P (rs73635825) [23]. ACE-2 polymorphisms alter expression of ACE-2 on cells and are predicted to affect affinity to RBD [22–25]. ACE2 polymorphisms S19P and K26R are predicted to confer increased susceptibility to infection due to increased binding affinity to ancestral SARS-CoV-2 RBD and full spike, as well as VOCS Alpha, Beta, and Gamma [25–27]. In contrast, the ACE2 polymorphism E35K has decreased binding to RBD constituting a more protective phenotype [25–27]. However, the impact of RBD mutations of VOCs, namely Delta and Omicron, on the affinity to these protein-altering ACE2 polymorphisms is yet to be characterized.

With the rise of VOCs, it remains crucial to understand the impact of RBD mutations on key facets of host interactions that may contribute to SARS-CoV-2 infection dynamics. Neutralizing antibodies have been demonstrated to be important for protection against both ancestral SARS-CoV-2 and subsequent variants [28]. In addition, growing evidence suggest that Fc-mediated antibody responses contribute to protection and control of disease [15–19]. Importantly, Fc-mediated antibodies do not need to block specific neutralizing antibody epitopes, but can engage any viral target including conserved regions of the virus [29]. Thus here, our study comprehensively analyzes the impact of RBD mutations on IgG binding, ACE2 inhibition, and FcγR binding—from the plasma of BNT162b2-vaccine recipients and mild-to-moderate convalescent donors using multiplexing. We show a loss of IgG recognition against the RBD of VOCs, notably Omicron and Beta, and numerous single-point mutations, including G446S and N501T as compared to wild-type RBD. This reduction translates to a loss of FcγR-binding antibodies, further validated using ADCP assays. Interesting, while we observed a significant loss in ACE2-binding inhibition against Omicron, FcγR-binding antibodies—although decreased compared to wild type—were better retained than ACE2 inhibition, which suggested a retained potential to recruit Fc functions. Additionally, we provide evidence that affinity between ACE2 and RBD influences the potential for antibodies to block ACE2-binding, using a surrogate in vitro neutralization assay. Using biolayer interferometry, we demonstrate that mutations found in VOCs and human ACE2 polymorphisms (E35K, K26R, S19P), alter the affinity and binding kinetics between RBD and ACE2, which may ultimately influence an individual’s susceptibility to COVID-19 infection. With the RBD–ACE2 interface a critical aspect of viral entry, our data highlight key facets of the host antibody response and viral interactions that are modulated by mutations, that may have downstream implications for viral fitness.

## Methods

### Human samples and ethics statement

Convalescent COVID-19 plasma samples were collected from individuals with mild-to-moderate disease as previously described (*n* = 15) [30]. Briefly, these samples were collected early-2020 (March to May) during the first wave in Australia and were likely infected with the ancestral strain. Vaccine plasma samples were obtained from individuals prior to vaccination (baseline) and 2 weeks following second dose with Pfizer-BioNTech (BNT162b2) (*n* = 16) as previously described [31]. Participants characteristics for convalescent COVID-19 subjects and vaccine recipients are detailed in Table 1. Whole blood was collected into sodium heparin anticoagulant coated vacutainers before plasma was collected and stored at − 80 ℃. Study protocols were approved by the University of Melbourne Human Research Ethics Committee (#2056689).

**Table 1:** Characteristics of study cohort

	Baseline (*n* = 16)	BNT162b2 recipients (*n* = 16)	COVID-19 patients (*n* = 15)
Age, median (IQR), years	30.5 [5.5]	30.5 [5.5]	49 [23]
Gender			
Female, *n* (%)	10 (62.5%)	10 (62.5%)	9 (60%)
Male, *n* (%)	6 (37.5%)	6 (37.5%)	6 (40%)
Days from symptom onset, median (IQR)			38 [10.5]
Severity			
Mild, *n* (%)			9 (60%)
Moderate, *n* (%)			6 (40%)
BNT162b2-vaccine			
Days since second dose, median (IQR)		13 [0.75]	

*Mild defined as upper respiratory tract symptoms only; Moderate defined as including lower respiratory track symptoms. All patients non-hospitalized

### Proteins

SARS-CoV-2 antigens of the ancestral wild-type virus (B.1) are as follows: spike S1 (Sino Biological; 40,591-V08H), spike S2 (ACRO Biosystems; S2N-C52H5), nucleoprotein (ACRO Biosystems; NUN-C5227) and spike trimer (provided by Adam Wheatley) [32]. Influenza H1Cal2009 (Sino Biological; 11,085-V08H) and SIVgp120 (Sino Biological; 40,415-V08H) were used as positive and negative controls, respectively (See Supplementary Table 1).

#### RBD recombinant proteins

SARS-CoV-2 RBD recombinant proteins, listed in Supplementary Table 2, were synthesized as previously detailed [33]. Briefly, the sequences (obtained from GISAID surveillance repository) of the ancestral strain and RBD mutants were subcloned into pcDNA3.4 vectors by GenScript Corporation and subsequently expressed in Expi293 HEK cells [30]. All RBD proteins underwent a two-step purification process, initially through HisTrap Excel HP columns (Cytiva) followed by a Superdex 75 Increase 10/300 pg gel filtration column platform (GE Healthcare), before protein concentrations were determined (absorbance measurement wavelength at 280 nm).

#### hACE2

Synthesis of truncated human ACE2 (residues 19–613 with a C-terminal AviTag and 6xHis-tag) was conducted as previously described [33]. Targeted biotinylation was then performed using BirA enzyme prior to purification by strong anion exchange (Cytiva) [33].

### Multiplex platform

#### Coupling of protein antigens to beads

Custom Luminex multiplex arrays were established to study SARS-CoV-2 antibody responses in accordance with the previously established protocols. A complete list of protein antigen quantities-to-bead ratio for the *SARS-CoV-2-specific multiplex assay* and *ACE2-inhibition assay* can be found in Supplementary Table 1 and Supplementary Table 2, respectively.

#### SARS-CoV-2-specific multiplex assay

To characterize antibody responses across plasma samples, we utilized a broad panel of SARS-CoV-2 antigen-coupled beads (see Supplementary Fig. 1) in a Luminex multiplex array as previously described [34]. Briefly, 1000 beads/bead region diluted in PBS containing 0.1% BSA (incubation buffer) were added to each well (in a total of 20 µl volume/well) in 384-well plates (Greiner Bio-One; 781,906), followed by addition of 20 µl/well of plasma diluted at a single concentration (1:100 in PBS). Plates were incubated on a plate shaker overnight at 4 ℃ before wells were washed with PBS 0.05% Tween-20 (PBST). Phycoerythrin (PE)-conjugated mouse anti-human antibodies that detect isotype (*Pan*-IgG, IgA1; Southern Biotech; 9040-09; 9130-09), or biotinylated IgM (MabTech; mAb MT22), or biotinylated Fc receptors (FcγRIIa-H131, FcγRIIIa-V158) (kindly provided by Bruce Wines and Mark Hogarth [35]) were diluted to 1.3 µg/ml and added at 25 µl volume/well before 2 h at room temperature on a plate shaker. For biotinylated detectors, plates were followed with an additional PBST wash, and incubated with streptavidin-PE (Invitrogen; S866) at room temperature for 1 h. Plates were then washed with PBST and each well was resuspended in 50 µl of sheath fluid. The level of PE signal associated with each bead region in each well, reported as median fluorescence intensity (MFI), was determined by a Flexmap3D Luminex platform. Each sample was run in duplicate.

#### ACE2-inhibition assay

We employed a recently established surrogate neutralization assay capable of simultaneously measuring the neutralizing capacity of antibodies against multiple RBD variants (See Supplementary Table 2), following a slightly modified version of the protocol [33]. Briefly, 700 beads/bead region diluted in incubation buffer were added to each well (in a total of 20 µl volume/well) in 384-well plates, followed by application of plasma, prepared as an 8-point serial dilution beginning at 1:5 in PBS for each sample (20 µl/well). Plates were covered and incubated on plate shaker for 1 h, and then 20 µl/well of biotinylated hACE2 diluted to 25 µg/ml was added, followed again by 1-h shaking at room temperature. Plates were then washed with PBST and incubated with 40 µl of streptavidin-PE (Invitrogen; S866) diluted to 4 µg/ml in incubation buffer on a plate shaker for 1 h. Following incubation, plates were incubated for an additional 1 h with 10 µl/well of R-PE Biotin-XX conjugate (Thermo Fisher Scientific; P811) diluted to 10 µg/ml before washing with PBST and resuspension in 50 µl of sheath fluid. Flexmap3D Luminex platform was used for acquisition of samples and reported as MFI for each bead region used. Plates were run in duplicate.

For wells used in the detection of isotype (IgG, IgM, IgA) and FcR-binding features of RBD-specific antibodies, 20 µl/well of incubation buffer-*only* was added in replacement of diluted-biotinylated hACE2. Plates were then washed with PBST and incubated with respective detectors in methods described above.

#### Omicron assays

Given that this study began before the emergence of the Omicron, to study Omicron responses, ancestral WT RBD and Omicron BA.2 RBD were purchased from Sino Biological and the above described ACE2 inhibition and multiplex assays were conducted in a separate assay.

### Bio-layer interferometry (BLI)

The WT ACE2–RBD variant binding kinetics Bio-layer interferometry (BLI) was performed on the Octet Red instrument (FortéBio) as previously described [33]. Briefly, Streptavidin Biosensors (FortéBio) hydrated in HBS-EP buffer ((0.01 M HEPES, 0.15 M NaCl, 3 mM EDTA, and 0.005% v/v Surfactant P20, pH 7.4 (GE Healthcare)) for 20 min were programmed to perform loading of biotinylated ACE2 at 3 µg/ml. Once the ACE-2 binding response reached a threshold of 1.6 nm, the sensors were equilibrated in buffer-*only* wells for 120 s (baseline signal). The ACE2-loaded sensors then captured respective RBD variants at concentrations ranging from 100 nM to 6.25 nM (diluted via twofold serial dilution) for 150 s (association phase). Sensors were finally immersed in buffer-*only* wells for 360 s to record the dissociation signal. Curve fitting was performed using a global fit 1:1 Langmuir binding model using Octet Data Analysis software v12.0.2.3 (FortéBio), and baseline drift was corrected by reference subtracting the shift of an ACE2-loaded sensor immersed in kinetic buffer only. Mean kinetic constant values from 2 independent experiments were determined, with all binding curves matching the theoretical fit with an *r*^2^ value of more than 0.99. The complex *t*_1/2_ in seconds was calculated using the formula: *t*_1/2_ = 1n2/kdis = approximately 0.69/ koff.

BLI for measuring ACE-2 polymorphism binding affinity to RBD variants utilized the same protocol as above. The difference is only at the association phase, where sensors loaded with respective ACE-2 variants at 3 µg/ml concentration were immersed into wells with RBD variants at a single concentration of 100 nM for 150 s. Baseline, loading, and dissociation phases were as described above. A local fitting 1:1 binding model was used to calculate the curve fit and kinetic constants.

Both assays were performed in 96-well plates (Greiner Bio-One™ Polypropylene 96-Well F-Bottom Microplates, black (Interpath 655,209) at 1000 rpm and 30 ℃.

### Bead-based ADCP duplex assay

To assay the capacity for vaccinee plasma to mediate ADCP to SARS-CoV-2 wild type and to VOCs, a competitive, sample sparing duplex ADCP assay was adapted from previously described assays [14, 36, 37]. SARS-CoV-2 RBD wild type (WT) and SARS-CoV-2 RBD derived from the VOC Beta (B.1.351) were biotinylated using EZ-Link Sulfo-NHS-LC biotinylation kit (Thermo Scientific) with 20 mmol excess according to the manufacturer’s instructions and buffer exchanged using 30 kda Amicon centrifugal filters (EMD millipore) to remove free biotin. Red or yellow–green 1 μm fluorescent NeutrAvidin Fluospheres beads (Invitrogen) were coupled overnight at 4 ℃ to RBD WT and RBD Beta, respectively (1 μl beads:3 μg antigen). Coupled beads were washed thrice with 2% BSA/PBS to remove excess antigen, diluted 1:100 and combined to form a bead cocktail. 10 μl of bead cocktail (9 × 10^5^ beads) was added to each well in a 96-well U-bottom plate with titrations of baseline and vaccinee plasma (1:100–1:777,600) and incubated for 2 h at 37 ℃. THP-1 monocytes (100,000 cells/well) were added to opsonized beads and incubated for 16 h under cell culture conditions. Cells were fixed with 2% formaldehyde and acquired by flow cytometry on a BD LSR Fortessa with a HTS. The data were analyzed using FlowJo 10.8.1 (See Supplementary Fig. 1 for gating strategy) and phagocytosis scores were calculated for each bead type (RBD WT and RBD Beta) as previously described using the formula: [%bead-positive cells × mean fluorescent intensity] [38]. To account for non-specific uptake of S-conjugated beads, the phagocytosis scores for each plasma sample were subtracted with that of the “no plasma” control or baseline plasma samples (matched SARS-CoV-2 naïve plasma collected prior to vaccination. ED_50_’s was calculated for each sample and assays were performed in duplicate.

### Statistics

#### Systems serology (LASSO PCA)

Antibody features were selected using a least absolute shrinkage and selection operator (LASSO) reduction method as previously described using MATLAB version 9.6 (including machine learning and statistical toolbox) (The MathWorks, Inc., Natick, MA) and Eigenvector PLS toolbox (Eigenvector, Manson, WA) [39]. In brief, data were first processed by right-shifting (to remove negative values) and log transformation (using equation log_10_ (*x* + 1)) prior to being normalized using *z*-scoring. LASSO feature reduction was then employed on *z*-scored data and performed 1000-times with tenfold cross validation. Using LASSO-selected features, unsupervised principal component analysis (PCA) was then performed.

#### Data analysis

Prism GraphPad version 9.0.2 (GraphPad Software, San Diego, CA) were used to develop graphs and perform statistical analysis as described in respective figure legends.

## Results

### Greater IgG and FcγR-binding antibodies to ancestral SARS-CoV-2 spike and RBD in BNT162b2 recipients compared to mild-to-moderate convalescence

Sixteen individuals vaccinated with BNT162b2 had plasma samples drawn before vaccination (baseline) and two weeks (median: 13 days; IQR: 0.75 days) following the second dose (Table 1). We also studied plasma from fifteen convalescent individuals (median: 38 days post symptom onset; IQR: 10.5 days) with mild-to-moderate COVID-19 disease. Importantly, convalescent samples were collected during the first wave of the pandemic (between March 2020 and May 2020 in Victoria, Australia), providing an opportunity to study responses in individuals infected by viruses that harbor less phylogenic deviation from the ancestral reference strain used in the BNT162b2 mRNA vaccine.

**Fig. 1:**
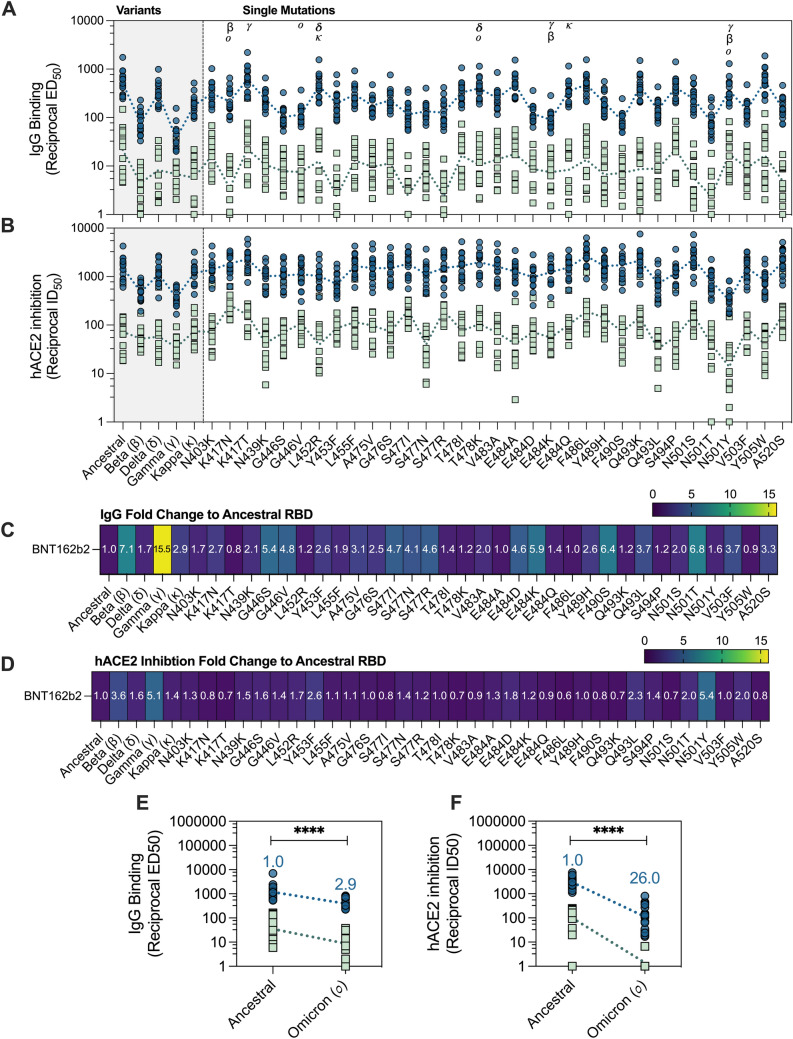
IgG binding and hACE2 inhibition to ancestral RBD and 38-RBD variants. **A** *Pan*-IgG binding levels (reciprocal ED_50_) and **B** human wild-type ACE2 (hACE2) inhibition levels (reciprocal ID_50_) by BNT162b2 (2 weeks following second dose; blue; *n* = 16) and mild/moderate convalescent COVID-19 patients (green; median 38 days post symptom onset; *n* = 15) as measured by multiplex to RBD variants. Reciprocal ED_50_/ID_50_ values, respectively, were calculated using the normalized binding MFI value for an 8-point serial plasma titration. **C** Fold change of *pan*-IgG binding levels of RBD variants in comparison to ancestral RBD and **D** Fold change of hACE2 inhibition of RBD variants in comparison to ancestral RBD by BNT162b2 plasma. Fold change was calculated as follows: (geometric mean of reciprocal ED_50_ or ID_50_ Ancestral RBD) divided by (geometric mean reciprocal ED_50_ or ID_50_ variant RBD). Friedman’s non-parametric test with Dunn’s multiple comparisons was used to assess statistical significance. **E** Pan-IgG binding levels (reciprocal ED_50_) and (F) hACE2 inhibition levels (reciprocal ID_50_) to RBD of Omicron BA.2 variant with fold change as indicated above in blue. Mann–Whitney *U*-test used for statistical evaluation. *p* < 0.05 (*), *p* < 0.01 (**), *p* < 0.001 (***) and *p* < 0.0001 (****)

First, plasma humoral profile to SARS-CoV-2 proteins (NP, RBD, S1, Trimer Spike) found in the ancestral wild-type virus was characterized using a SARS-CoV-2 multiplex bead assay. Robust antibody responses to NP were uniquely associated with convalescent COVID-19 individuals, indicating no prior infection in the BNT162b2-vaccinees (Supplementary Fig. 2). Convalescent and BNT162b2-vaccinees both elicited elevated levels of IgG to spike antigens; however as expected, BNT162b2-vaccinees demonstrated significantly greater responses in comparison to convalescent individuals which displayed more heterogenous responses (S1, 3.8-fold higher; trimer, 1,sixfold higher; RBD, 3.5-fold higher), (Fig. 1B–D; Supplementary Fig. 2). Next, we measured the capacity for antigen-specific antibodies to engage soluble FcγRIIa and FcγRIIIa-dimer constructs, which mimic the surface of innate immune cells and are a proxy readout for ADCP and ADCC activity, respectively. As before, plasma from BNT162b2-vaccinees showed significantly higher FcγRIIa and FcγRIIIa-dimer binding levels by anti-spike antibodies than mild-to-moderate convalescent patients (Supplementary Fig. 2; Supplementary Fig. 3). Notably IgG1 subclass levels strongly correlated with both FcγRs (FcγRIIa-H131: Pearson *r* = 0.94, *p* < 0.0001; FcγRIIa-H131: Pearson *r* = 0.90, *p* < 0.0001, Supplementary Fig. 3j). Unsupervised principal component analysis (PCA), used to broadly examine this repertoire of antibody responses, showed that baseline, BNT162b2 (2 weeks post dose two), and convalescent (mild-to-moderate) subjects were naturally separated into three distinct groups based on their antibody signatures (Supplementary Fig. 2E). This analysis confirms that BNT162b2 vaccination induces more robust anti-ancestral spike IgG responses with greater FcγR-binding capacity, in comparison to our mild-to-moderate convalescent donors.

### Reduced IgG-binding antibodies to SARS-CoV-2 RBD variants, but not always concordant to loss in ACE2 inhibition

The RBD is a mutational hotspot for SARS-CoV-2. Because of its position at the ACE2 interface, mutations in the RBD can alter ACE2 affinity as well as result in the loss of epitopes for antibodies [2, 40, 41]. Thus, to characterize the impact of RBD mutations on plasma IgG binding in our convalescent and BNT162b2 donors, we generated a custom panel of 39 RBDs, including RBDs of 33 common-point mutations, 5 variants of concern/interest (Beta, Delta, Gamma, Kappa, and Omicron) and the ancestral RBD, and measured antibody responses using multiplex. Higher levels of IgG binding responses (larger reciprocal ED_50_ values) were observed in BNT162b2-vaccines compared to convalescent individuals across all RBDs assessed (Fig. 1A, E), consistent with our previous observation that BNT162b2 elicits more robust anti-spike IgG responses. Despite the overall greater levels of IgG in vaccinees, significantly reduced IgG recognition was observed against the VOCs Beta (7.1-fold reduction, *p* < 0.0001) and Gamma (15.5-fold reduction, *p* < 0.0001) by BNT162b2 plasma in comparison to the ancestral RBD (Fig. 1C). Drop in IgG binding was also observed with Omicron (BA.2) (2.9-fold reduction, *p* < 0.0001) (Fig. 1E). Furthermore, significant decreases in IgG recognition to various point mutations, including G446S (5.4-fold reduction, *p* < 0.0001) found in Omicron, E484K (5.9-fold reduction, *p* < 0.0001) found in Gamma and Beta, as well as F490S (5.4-fold reduction, *p* < 0.0001) and N501T (6.8-fold reduction, *p* < 0.0001) (Fig. 1C). These data highlight the influence of particular point mutations on recognition of the RBD by IgG antibodies.

Since the RBD is a dominant target for neutralizing antibodies, we next considered the impact of these RBD variants on blocking ACE2 binding, using a surrogate neutralization assay. Using a previously established in vitro ACE2 inhibition assay, we measured the capacity for BNT162b2 and convalescent plasma to block hACE2 binding in a competitive format. Overall, the capacity for BNT162b2 plasma to block ACE2 binding was greater (larger reciprocal ID_50_ values) in comparison to convalescent plasma across all 39 RBDs (Fig. 1B, F). Consistent with the loss of IgG binding, we observed decreased ACE2 inhibition to all assessed VOC, with a significant decrease observed for Beta (3.6-fold reduction) and Gamma (5.1-fold reduction), though this reduction was notably smaller than that for IgG binding (Fig. 1C). A substantial drop in ACE2 inhibition was observed with Omicron (BA.2) (26-fold reduction; *p* < 0.0001) highlighting the evasive nature of this variant (Fig. 1F). On the other hand, most of the RBD point mutations which displayed reduced IgG binding did not necessarily translate to a significant drop in ACE2 inhibition (Fig. 1C, D). While a decrease in IgG binding was observed for the RBD mutations G446S and N501T, the loss in ACE2 inhibition observed was much weaker (G446S = 1.6-fold reduction, *p* = ns; N501T = 2.0-fold reduction, *p* = 0.0013) (Fig. 1D; Supplementary Table 3). A 1.7-fold reduction (*p* = 0.04) in ACE2-blocking was also observed for L452R, despite having comparable IgG binding (*p* = ns) to the ancestral RBD. Furthermore, the RBD mutation N501Y, the prime mutation found in the RBD Alpha-VOC, demonstrated no significant decrease in IgG binding (1.6-fold reduction, *p* > 0.99) but showed a significant drop in capacity to block ACE2 from binding (5.4-fold reduction, *p* < 0.0001) (Fig. 1C, D; Supplementary Table 3). Together, these data highlight the influence of naturally occurring RBD mutations on the potential for polyclonal antibodies to recognize and block ACE2 binding.

### SARS-CoV-2 VOC RBDs have improved affinity to WT hACE2

**Fig. 2:**
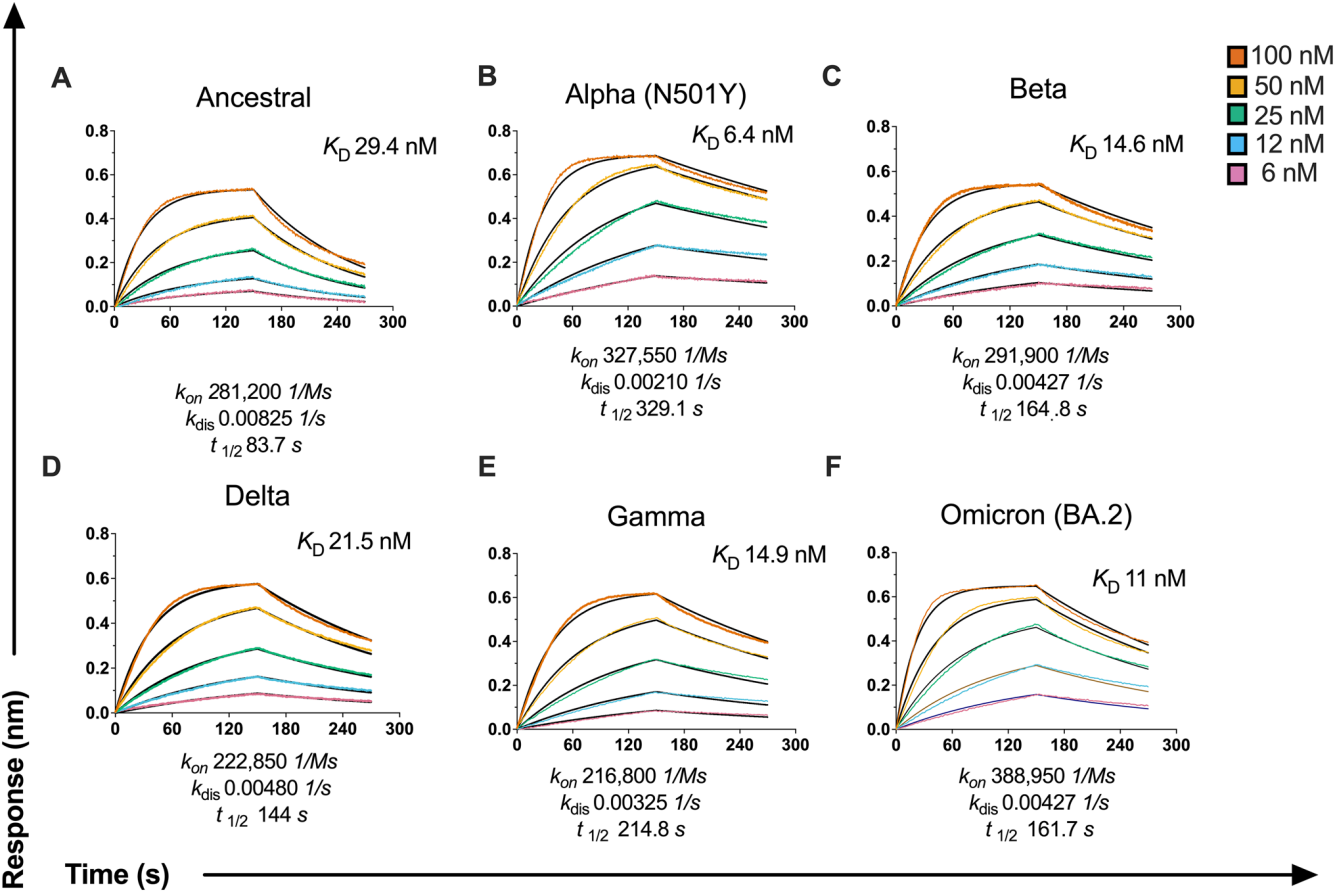
Affinity of wild-type (WT) hACE2 to the RBD of variants. Sensograms of WT-human ACE2 (hACE2) binding affinity to **A** ancestral RBD, **B** Alpha (B.1.1.7), **C** Beta (B.1.351), **D** Delta (B.1.617.2) **E** Gamma (P1), **F** Omicron (BA.2) as measured by biolayer interferometry (BLI). Binding of twofold serial dilutions of each SARS-CoV-2 RBD variant (100 nM (red), 50 nM (yellow), 25 nM (green), 12 nM (blue), and 6 nM (pink)) was measured to immobilized hACE2 (3 μg/ml). Binding curves representative of 2 independent experiments for each variant are plotted (solid-colored lines), globally fitted to a 1:1 binding model (black line)

Mutations within the RBD also have the potential to modulate affinity to hACE2 [2, 4, 41, 42]. Hence, we next characterized the binding affinity of wild type (WT)-ACE2 to VOC RBDs, as well as a subset of single-point mutations, using biolayer interferometry (Fig. 2; Supplementary Fig. 4). We found all VOC RBDs assessed demonstrated elevated binding affinity to WT ACE2 in comparison to ancestral RBD (*K*_d_: 29.4 nM; *t*_1/2_: 83.6 s) (Fig. 2). These included Beta (*K*_d_: 14.6 nM; *t*_1/2_: 164.83), Gamma (*K*_d_: 15.0 nM; *t*_1/2_: 214.82 s) and Delta (*K*_d_: 21.5 nM; *t*_1/2_: 143.77 s). Omicron (BA.2) demonstrated a 2.6-fold increase in binding (*K*_d_: 11 nM; *t*_1/2_: 161.75 s), compared to ancestral RBD (Fig. 2). Together, these data reiterate how these mutations in the RBD, given its position at the ACE2 interface, can potentially improve viral fitness by enhancing affinity to ACE2. Furthermore, these data suggest that the RBD mutations observed in Beta and Gamma not only significantly reduced IgG binding, but also compounded with stronger ACE2 affinity to give the largest drops in ACE2 inhibition.

Previously, we and others have shown single-point mutations in RBD (such as N501Y found in Alpha, Beta, Gamma and Omicron) can enhance affinity to ACE2 (4.5-fold increase; *K*_d_: 6.5 nM; *t*_1.2_: 329.1 s, Fig. 2B) [4, 33, 40]. With N501Y, we observed a reduced capacity for antibodies to neutralize, despite no loss in total IgG (Fig. 1). Similar to N501Y, here we observed using BLI that while L452R did not differ greatly from the ancestral RBD in total IgG binding, it exhibited a stronger affinity (*K*_d_: 27.36 nM; *t*_1.2_: 126.4 s), which in turn could explain the drop in plasma-driven ACE2 inhibition (Supplementary Fig. 4; Supplementary Table 4). Likewise, using BLI, we observed that N501T had improved binding affinity (*K*_D_: 11.05 nM; *t*_1.2_: 217.8 s) as compared to the ancestral RBD (Supplementary Fig. 4; Supplementary Table 4). As such, the combination of reduced IgG binding due loss of epitope and stronger ACE2 affinity for N501T could further impeded ACE2 inhibition by plasma samples. Together, these results suggest that beyond antibody recognition, affinity interactions between RBD and ACE2 exert another layer of influence on the potential for antibodies to effectively block ACE2-binding and achieve neutralization.

### hACE2 polymorphisms alter affinity to SARS-CoV-2 RBD variants

**Fig. 3:**
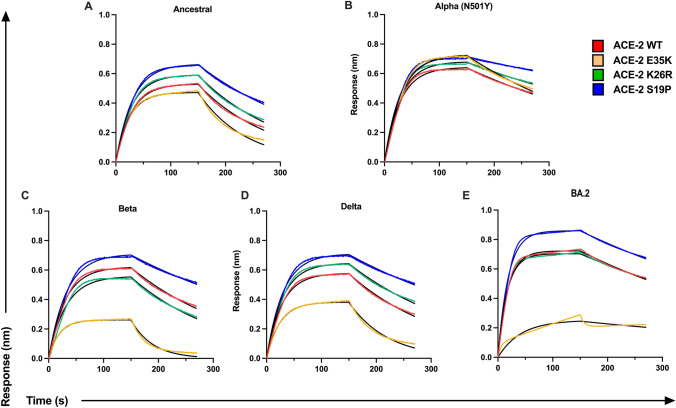
Affinity of hACE2 polymorphisms to VOC RBD’s. Binding profiles of WT hACE2 (purple) and three polymorphisms (E35K, green; K26R, red; S19P, blue) to **A** ancestral RBD and RBD of variants of concern **B** Alpha (B.1.1.7), **C** Beta (B.1.351), **D** Delta (B.1.617.2), **E** Omicron (BA.2). Sensograms show 100 nM of immobilized RBD of each variant run against 100 nm (3 μg/ml) ACE2 of each polymorphism to measure binding kinetics

Several ACE2 polymorphisms are present at low frequency within the human population, with certain alleles more prevalent in different populations (Supplementary Table 6). In particular, K26R has been observed at low frequencies across most populations, whereas low frequencies of S19P are predominantly detected within African/African American populations (0.3%) [22, 24]. Previous work has shown these polymorphisms confer altered affinity to ancestral RBD; however, the binding kinetics to emerged VOC RBD, especially Delta and Omicron, has yet to be described. Here, we assessed the binding profiles of three low-frequency ACE2 polymorphisms (E35K, K26R and S19P) to 5 VOC RBDs (Alpha, Beta, Delta, Gamma, Omicron (BA.2)) and ancestral RBD using biolayer interferometry (BLI). We found that the polymorphism S19P (*K*_D_: 14.38 nM) and K26R (*K*_D_: 20.0) demonstrated a 2.04-fold and 1.47-fold increase in binding affinity, respectively, to ancestral RBD when compared to WT ACE2 (*K*_D_: 28.99 nM) (Fig. 3, Supplementary Table 5). Furthermore, this enhanced affinity of the S19P and K26R polymorphisms remains overall elevated against the RBD’s of Beta (*K*_D_: S19P = 10.51 nM, K26R = 17.46 nM), Omicron (BA.2) (*K*_D_: S19P = 4.97 nM, K26R = 5.18 nM) and Delta (*K*_D_: S19P = 8.56, K26R = 13.47) (Fig. 3, Supplementary Table 5). These data suggest that individuals who harbor S19P or K26R ACE2 polymorphisms may have greater susceptibility to infection by SARS-CoV-2 due to increased ACE2 affinity. Contrastingly, ACE2 polymorphism E35K demonstrated weaker binding when compared to ancestral RBD (*K*_D_: 36.63 nM), Omicron (BA.2) (*K*_d_: 18.50 nM), and Delta (*K*_d_: 43.34 nM), suggesting that individuals harboring the E35K ACE2 polymorphism may demonstrate a more protective phenotype due to reduced ACE2 affinity for RBD (Fig. 3; Supplementary Table 5). Together, these data suggest that ACE2 polymorphisms, though low in frequency, can modulate binding to ACE2, and by extension may change an individual’s susceptibility to infection.

### FcγR-binding antibodies to RBD variants

**Fig. 4:**
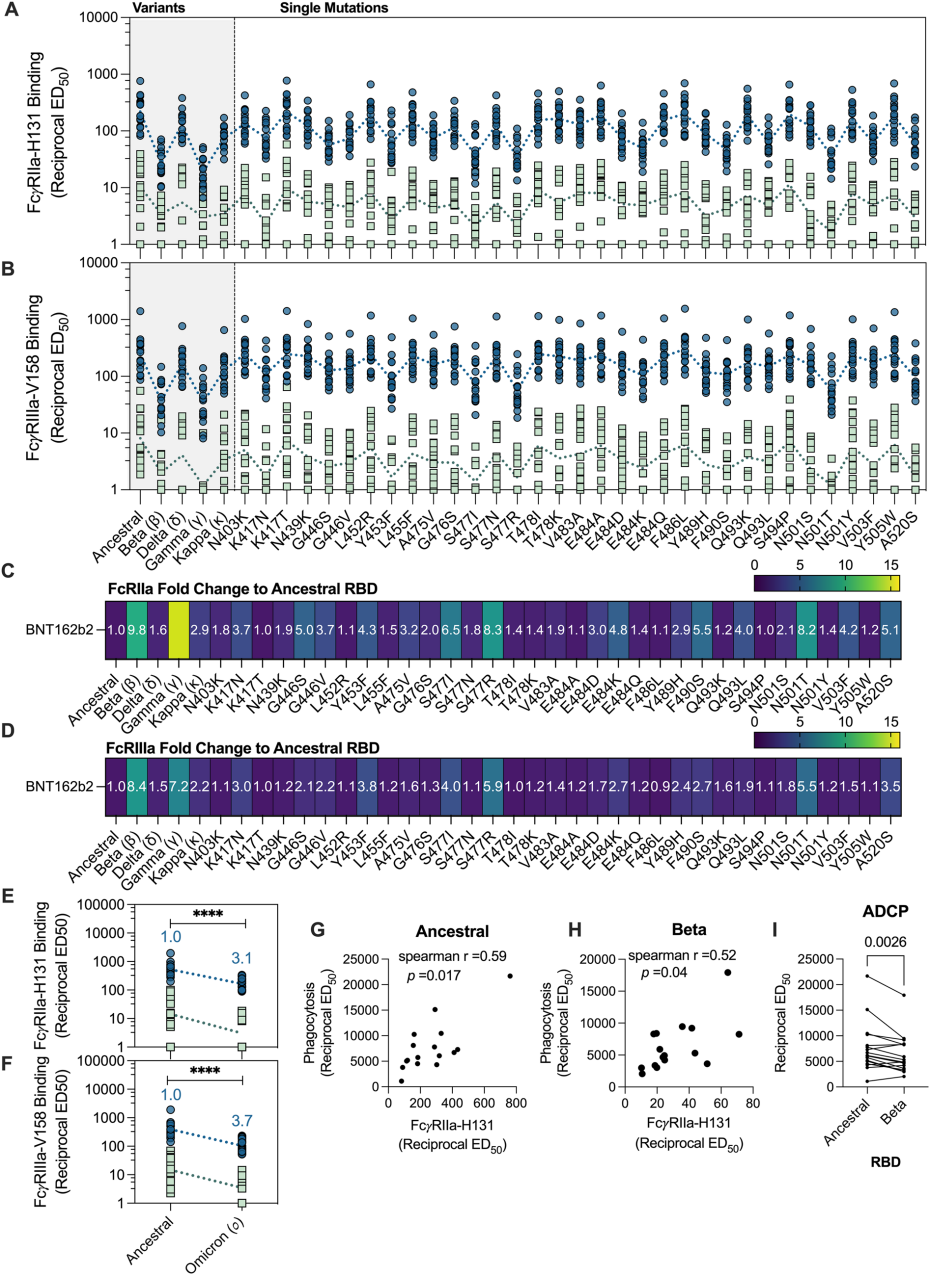
Loss of anti-RBD FcγR-binding antibodies and ADCP responses to VOC RBDs in BNT162b2 and convalescent plasma. **A** FcγRIIa-H131 and **B** FcγRIIIa-V158 binding levels of RBD-specific antibodies using plasma of BNT162b2 (2 weeks following second dose; blue; *n* = 16) and mild/moderate convalescent COVID-19 patients (green; median 38 days post symptom onset; *n* = 15) as measured via multiplexing. **C** Fold change of anti-RBD FcγRIIa-H131 binding levels to RBD variants in comparison to ancestral RBD of BNT162b2 plasma. **D** Fold change of FcγRIIIa-V158 levels of RBD variants in comparison to ancestral of BNT162b2 plasma. Reciprocal ED_50_ values were calculated using the normalized FcγR binding MFI value for an 8-point serial plasma titration. Fold change was calculated as follows: (geometric mean of reciprocal ED_50_ or ID_50_, respectively ancestral RBD) divided by (geometric mean reciprocal ED_50_ or ID_50_, respectively variant RBD). Friedman’s non-parametric test with Dunn’s multiple comparisons was used to assess statistical significance. **E** FcγRIIa-H131binding (Reciprocal ED_50_) and **F** FcγRIIIa-V158 inhibition levels to RBD of Omicron variant with fold change of BNT162b2 plasma indicated above in blue. Mann–Whitney U-test used to assess statistical significance. **G**, **H** Spearman correlations of phagocytosis activity (reciprocal ED_50_) using THP-1 monocytes and multiplex FcRIIa-H131 data (reciprocal ED_50_) against the ancestral and Beta RBD. **I** ADCP by THP-1 monocytes induced by the plasma of BNT162b2 against the ancestral and Beta RBD shown as phagocytic score ED_50_. Wilcoxon matched pairs signed rank test was used to assess statistical significance. *p* < 0.05 (*), *p* < 0.01 (**), *p* < 0.001 (***) and *p* < 0.0001 (****)

Several previous studies have highlighted the importance for Fc-effector functions during SARS-CoV-2 infection, yet to date, little work has widely characterized the impact of RBD mutations on FcγR-binding [14, 15, 17, 18, 20]. Given the observed loss of IgG to certain RBD variants, we, thus, next investigated the impact that these mutations conferred on the capacity to for anti-RBD antibodies to engage our FcγRIIa and FcγRIIIa-dimer constructs, a surrogate measure for ADCP and ADCC activity, respectively. We used our multiplex panel of 39-RBD to address the impact of these mutations on FcR-binding antibodies as compared to the ancestral RBD. Consistent with our previous observation to total IgG levels, BNT162b2 vaccinee plasma had a significantly greater capacity (higher reciprocal ED_50_ vales) to engage both our FcγRIIa and FcγRIIIa-dimer constructs across all variants, in comparison to convalescent plasma (Fig. 4A, B). Indeed, antigen-specific IgG levels highly correlated with FcγRIIa and FcγRIIIa-binding of their respective variants (Supplementary Fig. 5). Compared to ancestral RBD, VOC Beta and Gamma demonstrated a decrease in anti-RBD FcγRIIa (9.8- and 14.9-fold reduction respectively; *p* < 0.0001) and FcγRIIIa-dimer-binding antibodies (8.4-, and 7.2-fold reduction respectively; *p* < 0.0001) (Fig. 4C, D). Furthermore, we observed that single-point mutations with poor IgG binding, such as G446S, displayed a reduction in FcγR-binding as well (FcγRIIa: 6.2-, 5.0-fold reduction, respectively; FcγRIIIa 5.5-, 2.1-fold reduction, respectively) (*p* < 0.0001) (Fig. 4C, D; Supplementary Table 3). Likewise, a drop was observed to Omicron BA.2 with FcγRIIa binding, with a marginally larger decrease to FcγRIIIa observed (3.1- and 3.7-fold reduction, respectively; *p* < 0.0001) (Fig. 4E, F). Although interestingly, we noted this drop against Omicron was less pronounce than the decrease in ACE2 inhibition (26-fold reduction; *p* < 0.0001), suggesting FcγR-binding antibodies may be more resistant to mutations found in Omicron than ACE2 inhibition (Fig. 1F).

To validate our findings using the surrogate FcγR dimers, we next measured the capacity for BNT162b2 plasma to induced ADCP using a duplex bead-based assay with a THP-1 monocyte cell line. Phagocytic score reciprocal ED_50_ were calculated, with highly concordant values obtained when non-specific background responses was adjusted for using either a no plasma control or corresponding matched baseline samples (Pearson *r* = 0.98; *p* < 0.0001) (Supplementary Fig. 1D–F). FcγRIIa reciprocal ED_50_ values correlated well wit phagocytosis ED_50_ levels of ancestral RBD by THP-1 monocytes (spearman *r* = 0.59, *p* = 0.017), as well as the RBD of Beta (spearman *r* = 0.52, *p* = 0.04) (Fig. 4G, H). For both ancestral and beta RBD, IgG binding (reciprocal ED_50_) correlated strongly with FcγRIIa-dimer (Spearman *r* = 0.77 and 0.79, respectively; *p* = 0.0006 and 0.0004, respectively), and phagocytosis levels (Spearman *r* = 0.81 and 0.72, respectively; *p* = 0.0002 and 0.002, respectively) (Supplementary Fig. 6A–D). We observed a significant decrease in ADCP activity against ancestral vs Beta RBD (*p* = 0.0026), supporting the mutations found in VOCs are detrimental to FcγR-binding antibodies (Fig. 4I). Altogether, our data suggest that a reduced capacity for antibodies to induce Fc-effector functions against the RBD variants compared to RBD of wild type. However, in the context of Omicron, FcγR-binding antibodies be more readily maintained than ACE2 inhibiting antibodies.

## Discussion

The emergence of multiple SARS-CoV-2 variants has led to new waves of infection across the globe, with the RBD of SARS-CoV-2 spike being a key site for escape mutations [1, 43]. Here, we showed reduced recognition of RBD variants by IgG plasma antibodies from both BNT162b2 and convalescent subjects, with profound loss against VOCs Beta, Gamma, and more recent Omicron, indicative of evolutionary immune escape. Using our surrogate RBD–ACE2 multiplex bead assay, we showed that the drop in IgG binding against VOCs corresponded to a reduction in ACE2 inhibition, consistent with others [44–46]. Furthermore, in line with loss of IgG binding, we also found a decrease in anti-RBD FcγR -binding antibodies against variants, suggesting FcγR functions were decreased against VOC RBDs compared to wild type. This supports recent work that also observed that variants with larger number of mutations, e.g., Omicron induce lower FcγR functions compared to earlier VOCs with less mutations [47]. Intriguingly, this study also observed reduced FcγR functions specifically to Omicron BA.2 as compared to BA.1, suggesting that FcγR functions may be uniquely influenced by certain mutations [47]. Bartsch et al. also went on to show that FcγR-functions against the full-VOC spike are less drastically impacted by mutations, likely reflecting the greater number of conserved epitopes harbored on the full-spike that can be targeted by both neutralizing and non-neutralizing antibodies [48]. Intriguingly, our findings show that the erosion of ACE2 inhibition against the RBD of Omicron (BA.2) by BNT162b2 plasma was comparably larger than the loss of FcγR binding, suggesting that many non-neutralizing epitopes on the Omicron RBD may remain conserved despite the multiple (> 16) point mutations that are otherwise detrimental for neutralization. This supports recent murine mRNA SAR-CoV-2 vaccine studies which observed that neutralizing antibody responses induced by vaccination alone were not sufficient for protection and that Fc receptor responses are essential for protection against Omicron [21].

A key finding in our study was that human ACE2 polymorphisms, whilst found in low frequencies, demonstrated distinct variation in affinity to the RBD. All VOC RBDs (Beta, Gamma, Delta, Omicron (BA.2) bind WT ACE2 with enhanced affinity, reflecting evolutionary mutations that enhance viral entry and transmission [27, 42]. However, here, we provided new insights into the binding affinity of VOC- RBDs, notably Delta and Omicron, to several ACE2 N-terminal polymorphisms—the site for RBD docking. K26R (rs4646116),—the second most frequent human ACE2 polymorphism (0.4% allele frequency), displayed enhanced binding affinity to ancestral RBD, and VOCs, Alpha, Delta, while maintaining similar affinity to Omicron [25, 49]. S19P which is predominantly observed within African/African America populations (0.3%) also showed enhanced affinity to ancestral RBD, along with all tested VOC (Alpha, Beta, Delta, and Omicron). These stronger binding affinities to VOCs RBDs may pre-dispose subsets of individuals harboring these polymorphisms to become more susceptible to breakthrough infections by SARS-CoV-2 variants [22, 23, 25, 49–51]. Intriguingly, K26R (rs4646116) demonstrated slightly weaker binding to RBD Beta. E35K (rs1348114695, which has been predominantly detected at very low frequencies (0.01%) in East Asian populations) also had decreased affinity to ancestral RBD and more importantly demonstrated an even greater loss in binding affinity to VOC RBDs, particularly Beta, Delta, and Omicron, which may confer some level of natural protection against breakthrough infections by these variants. Our work suggests that large genetic linkage studies together with VOC infection and severity data could be informative.

Assessing single-point mutations found in the RBD can provide valuable insights into the importance of these mutations on overall viral fitness. Our study showed numerous point mutations imparted little impact on anti-RBD IgG recognition as well as FcγR-binding engagement. This was observed for RBD point mutations L452R and T478K (both found in Delta and several Omicron sub-variants), which may account for the limited reduction in total IgG binding and FcγR-binding observed against Delta RBD. However, we noted that L452R-RBD had a longer binding half-life to WT ACE2, which could contribute to the observed loss of ACE2 neutralization. Indeed, it has been described that L452R increases SARS-CoV-2 infectivity and fusogenicity and, thus, may confer an evolutionary advantage previously observed with Delta and, more recently, the Omicron sub-lineages BA.4 and BA.5 [52]. On the other hand, certain point mutations, such as G446S (found in Omicron), G446V, S477I, and S477R, conferred reduced recognition by IgG. This suggests that mutations at amino-acid positions 446 and 477 are advantageous for viral immune escape, which is further reinforced by loss of FcγR-binding. Interestingly, while S477N (found in Omicron) also resulted in a drop in total IgG binding, it was less disruptive to FcγR-dimer engagement than both S477I and S477R [25, 26]. We also observed the largest fall in total IgG binding against N501T, which displayed increased ACE2 affinity and longer binding half-life resulting in a significant loss in neutralization. While N501T had weaker ACE2-binding affinity compared to N501Y—a key RBD mutation found previously in several VOCs including Omicron—the loss in FcγR binding was more profound with N501T, corresponding to the drastic reduction in total IgG binding. This is of concern for zoonotic transmission as N501T is a dominant RBD mutation identified in SARS-CoV-2-infected minks, ferrets, and deer [53, 54].

Several future studies are suggested by our work. While our cohort of BNT162b2-individuals received two doses of vaccine, the current approved regimen is now expanded to three doses in Australia and many other countries. Three doses enhance antibody responses to VOCs overall and it will be important to dissect particular mutations for their impact on improved RBD-specific antibody responses. Interestingly, Irrgang et al., recently reported enhanced class-switching to IgG4 following repeated BNT162b2 vaccination associated with reduced Fc functional activity [55]. While this study demonstrated that IgG4 reduced Fc functions to ancestral SARS-CoV-2, it remains unclear whether elevated IgG4 levels also influence Fc activity to different SARS-CoV-2 VOCs. Furthermore, neutralizing antibody responses elicited from current Omicron BA.2 infections have been shown to differ from that of earlier waves (Omicron BA.1 and Delta), and this will also likely impact the specificity of RBD responses [56]. Omicron viruses have further evolved past BA2, and there are now additional lineages with BA.4. and BA.5 currently in circulation and XBB1.5 currently classified as a Variant of Interest by the WHO. Thus, future studies will aim to examine immune escape and ACE2 affinity to these variants. Research in other viral infections, such as RSV, has observed antibody-dependent enhancement (ADE) of infection in vitro*,* when low neutralizing antibody titers but competent Fc activity is retained [57]. Conversely in other viral infections such as HIV and Ebola, retained Fc-effector functions have been demonstrated to be essential for improved protection or control of infection in vivo, especially at suboptimal neutralizing antibody titers [58–60]. Importantly, no study to date has observed ADE antibodies following SARS-CoV-2 vaccination, in vivo [61].

Overall, our data show that RBD mutations, both common point mutations as well as combinations of mutations in VOCs, not only have an impact on affinity to receptor ACE2, but also IgG binding and the Fc functional antibody response, thereby providing an important site for viral adaptions.

## Supplementary Information

Below is the link to the electronic supplementary material.

Supplementary file1 Supplementary Figure 1. Schematic representation of duplex phagocytosis assay with gating strategy and reciprocal ED50 analysis. (A) Schematic of the antibody dependent phagocytosis (ADCP) duplex assay. Briefly, APC fluorescent beads coated with ancestral RBD, while FITC fluorescent beads coated with RBD Beta were added to wells and incubated with plasma for 2 hours at 37OC. Following incubation, THP-1 monocytes were added to wells and incubated with opsonised beads for 2 hours under cell culture conditions before fixing cells for flow cytometry. (B) Representative gating strategy using pooled 2-week post vaccination plasma (blue), pooled baseline plasma (red) and no plasma (grey). Gating was performed by gating on THP-1 monocytes, single cells and finally cells positive for RBDWT beads and RBD Beta beads. (C) Visualization of single and double bead positive cells as dot plots for representative samples. ADCP by THP-1 monocytes induced by the plasma of BNT162b2 (two-weeks following second dose; n = 16) against the ancestral and Beta RBD shown as phagocytic score reciprocal ED50. Reciprocal ED50 values of ADCP duplex assay (D) without baseline correction using no plasma control (no baseline correction) and (E) with baseline correction using matched plasma samples collected prior to vaccination (baseline correction). (F) Pearson correlation of ED50 values calculated with no baseline correction and baseline correction. Supplementary Figure 2. SARS-CoV-2 serological signatures distinguish BNT162b2 vaccinated, convalescent, and baseline individuals. Plasma humoral responses were profiled via multiplex for convalescent mild/moderate COVID-19 patients (green square; median 38 days post symptom onset; n = 15) and BNT162b2-vaccinated individuals at baseline (purple triangle; n = 16) and two-weeks following second dose (blue circle; n = 16). Radar plots show the median MFI (median fluorescence intensity) value of each antibody signature (IgG, IgA, IgM, IgG1-IgG4, FcγR-dimer binding) to NP (A), trimeric spike (B), S1 (C), and RBD (D). The median MFI for each feature was normalised using z-scoring prior to plotting. (E) Unsupervised principal component analysis comparing convalescent COVID-19 individuals and BNT162b2-vaccinated individuals at baseline and two-weeks following second dose of 15-SARS-CoV2 antibody features selected by a dimensionality reduction method (LASSO). 95% confidence intervals for each group are depicted by a coloured eclipse. (F) PC1 and PC2 loadings of LASSO selected features for NP (yellow triangle), trimeric spike (red circle), spike subunit-1 (S1) (orange inverted triangle) and receptor binding domain (RBD) (brown square) of the PCA. Supplementary Figure 3. IgG subclass recognition of SARS-CoV-2 antigens and correlations to FcγR engagement. Plasma antibody isotype response (pan IgG, IgA1, IgM) (A-C), IgG subclass responses (IgG1, IgG2, IgG3 IgG4) (D-G) and FcγR engagement (FcγR3av and FcγR2aH) (H-I) to NP, RBD, S1 and spike trimer were profiled via multiplex. Convalescent mild/moderate COVID-19 patients (green square; median 38 days post symptom onset; n = 15) and BNT162b2-vaccinated individuals at baseline (purple triangle; n = 16) and two-weeks following second dose (blue circle; n = 16) were assessed. (J) Pearson R correlation matrix of IgG subclass binding (IgG1, IgG2, IgG3, IgG4) and FcγR engagement (FcγR3aV and FcγR2aH) with R values in bold and P-values described in italics. Supplementary Figure 4. Affinity of wild-type (WT) hACE2 to the RBD mutations. Sensograms of WT-human ACE2 (hACE2) binding affinity to (A) K417N, (B) K417T, (C) G446S, (D) L452R (E) L452R E484Q, (F) Y453F, (G) S477N, (H) T478K, (I) E484D, (J) E484K and (K) 484Q , (L) S494P and (M) N501T as measured by biolayer interferometry (BLI). Binding of 2-fold serial dilutions of each SARS-CoV-2 RBD variant (100 nM (red), 50 nM (yellow), 25 nM (green), 12 nM (blue), and 6 nM (pink)) was measured to immobilised hACE2 (3μg/ml). Binding curves representative of 2 independent experiments for each variant are plotted (solid-colored lines), globally fitted to a 1:1 binding model (black line). Supplementary Figure 5. IgG-binding correlates with FcγRIIa-H131 and FcγRIIIa-V158-dimer binding. BNT162b2 (two-weeks following second dose; blue; n = 16) and mild/moderate convalescent COVID-19 patients (green; median 38 days post symptom onset; n = 15) as measured by multiplex to RBD variants. Reciprocal ED50/ID50 values respectively were calculated using the normalized binding MFI-value for an 8-point serial plasma titration. Spearman correlations of multiplexed (A) FcRIIa-H131 data (reciprocal ED50) and (B) FcRIIIa-V158 dimer-binding data (reciprocal ED50) against IgG (reciprocal ED50) against ancestral RBD and variant RBD. All corresponding p values were below 0.005. Supplementary Figure 6. IgG-binding correlates with ADCP. Spearman correlations of IgG-binding (reciprocal ED50) with multiplex FcRIIa-H131 data (reciprocal ED50) of (A) Ancestral and (C) Beta RBD. Spearman correlations of IgG-binding (reciprocal ED50) phagocytosis activity (reciprocal ED50) using THP-1 monocytes against (B) ancestral and (D) Beta RBD. Supplementary Table 1 Antigens used in SARS-CoV-2-specific array. Supplementary Table 2 RBD recombinant proteins (variants and single point mutations) used in RBD array. Supplementary Table 3 Friedmans Test using Dunn’s multiple comparisons statistical values for comparison of IgG-binding, ACE2-inhibition, FcRIIa-binding and FcRIIIa-binding of RBD-variants to ancestral RBD. Supplementary Table 4. Affinity constants of wild-type (WT) hACE2 to RBD with single point mutations. Measured via BLI, calculated from 2 independent experiments. Supplementary Table 5. Affinity of human ACE2 polymorphisms to SARS-CoV-2 RBD Variants. Measured via BLI, calculated from 2 independent experiments. Supplementary Figure 6. Frequency of human ACE2 polymorphisms (E35K, K26R, S19P) found within human populations.  Polymorphism data collated from Genome Aggregation Database (gnomAD; https://gnomad.broadinstitute.org) (PPTX 3590 KB)

## Data Availability

All relevant data are available in the manuscript or in the supplementary materials. Any additional supporting data can be provided upon request.

## References

[1] Magazine N (2022). Mutations and evolution of the SARS-CoV-2 spike protein. Viruses.

[2] Mannar D (2021). Structural analysis of receptor binding domain mutations in SARS-CoV-2 variants of concern that modulate ACE2 and antibody binding. Cell Rep.

[3] Jackson CB (2022). Mechanisms of SARS-CoV-2 entry into cells. Nat Rev Mol Cell Biol.

[4] Tian F (2021). N501Y mutation of spike protein in SARS-CoV-2 strengthens its binding to receptor ACE2. Elife.

[5] Khoury DS (2021). Neutralizing antibody levels are highly predictive of immune protection from symptomatic SARS-CoV-2 infection. Nat Med.

[6] Feng S (2021). Correlates of protection against symptomatic and asymptomatic SARS-CoV-2 infection. Nat Med.

[7] Torresi J (2022). A complementary union of SARS-CoV2 natural and vaccine induced immune responses. Front Immunol.

[8] Planas D (2021). Sensitivity of infectious SARS-CoV-2 B. 1.1. 7 and B. 1.351 variants to neutralizing antibodies. Nat Med.

[9] Planas D (2021). Reduced sensitivity of SARS-CoV-2 variant Delta to antibody neutralization. Nature.

[10] Alter G (2021). Immunogenicity of Ad26. COV2. S vaccine against SARS-CoV-2 variants in humans. Nature.

[11] Jangra S (2021). SARS-CoV-2 spike E484K mutation reduces antibody neutralisation. Lancet Microbe.

[12] Yu J (2022). Neutralization of the SARS-CoV-2 Omicron BA. 1 and BA. 2 variants. N Engl J Med.

[13] Natarajan H (2021). Markers of polyfunctional sars-cov-2 antibodies in convalescent plasma. MBio.

[14] Lee WS (2021). Decay of Fc-dependent antibody functions after mild to moderate COVID-19. Cell Rep Med.

[15] Yamin R (2021). Fc-engineered antibody therapeutics with improved anti-SARS-CoV-2 efficacy. Nature.

[16] Schäfer A (2021). Antibody potency, effector function, and combinations in protection and therapy for SARS-CoV-2 infection in vivo. J Exp Med.

[17] Tauzin A (2021). A single dose of the SARS-CoV-2 vaccine BNT162b2 elicits Fc-mediated antibody effector functions and T cell responses. Cell Host Microbe.

[18] Winkler ES (2021). Human neutralizing antibodies against SARS-CoV-2 require intact Fc effector functions for optimal therapeutic protection. Cell.

[19] Ullah I (2023). The Fc-effector function of COVID-19 convalescent plasma contributes to SARS-CoV-2 treatment efficacy in mice. Cell Rep Med.

[20] Chan CE (2021). The Fc-mediated effector functions of a potent SARS-CoV-2 neutralizing antibody, SC31, isolated from an early convalescent COVID-19 patient, are essential for the optimal therapeutic efficacy of the antibody. PLoS One.

[21] Mackin SR (2023). Fc-γR-dependent antibody effector functions are required for vaccine-mediated protection against antigen-shifted variants of SARS-CoV-2. Nat Microbiol.

[22] Singh H (2021). ACE2 and TMPRSS2 polymorphisms in various diseases with special reference to its impact on COVID-19 disease. Microb Pathog.

[23] Bosso M (2020). The two faces of ACE2: the role of ACE2 receptor and its polymorphisms in hypertension and COVID-19. Mol Ther-Methods Clin Dev.

[24] Stawiski EW (2020). Human ACE2 receptor polymorphisms predict SARS-CoV-2 susceptibility. BioRxiv.

[25] Suryamohan K (2021). Human ACE2 receptor polymorphisms and altered susceptibility to SARS-CoV-2. Commun Biol.

[26] Calcagnile M (2021). Molecular docking simulation reveals ACE2 polymorphisms that may increase the affinity of ACE2 with the SARS-CoV-2 Spike protein. Biochimie.

[27] Barton MI (2021). Effects of common mutations in the SARS-CoV-2 Spike RBD and its ligand, the human ACE2 receptor on binding affinity and kinetics. Elife.

[28] Cromer D (2022). Neutralising antibody titres as predictors of protection against SARS-CoV-2 variants and the impact of boosting: a meta-analysis. Lancet Microbe.

[29] Arnold KB, Chung AW (2018). Prospects from systems serology research. Immunology.

[30] Juno JA (2020). Humoral and circulating follicular helper T cell responses in recovered patients with COVID-19. Nat Med.

[31] Selva KJ (2021). Tear antibodies to SARS-CoV-2: implications for transmission. Clin Transl Immunol.

[32] Wheatley AK (2021). Landscape of human antibody recognition of the SARS-CoV-2 receptor binding domain. Cell Rep.

[33] Lopez E (2021). Simultaneous evaluation of antibodies that inhibit SARS-CoV-2 variants via multiplex assay. JCI Insight.

[34] Selva KJ (2021). Systems serology detects functionally distinct coronavirus antibody features in children and elderly. Nat Commun.

[35] McLean MR (2017). Dimeric Fcγ receptor enzyme-linked immunosorbent assay to study HIV-specific antibodies: a new look into breadth of Fcγ receptor antibodies induced by the RV144 vaccine trial. J Immunol.

[36] Ackerman ME (2011). A robust, high-throughput assay to determine the phagocytic activity of clinical antibody samples. J Immunol Methods.

[37] Butler AL, Fallon JK, Alter G (2019). A sample-sparing multiplexed ADCP assay. Front Immunol.

[38] Darrah PA (2007). Multifunctional TH1 cells define a correlate of vaccine-mediated protection against *Leishmania major*. Nat Med.

[39] Chung A (2015). Dissecting polyclonal vaccine-induced humoral immunity against HIV using systems serology. Cell.

[40] Chakraborty S (2022). E484K and N501Y SARS-CoV 2 spike mutants Increase ACE2 recognition but reduce affinity for neutralizing antibody. Int Immunopharmacol.

[41] Ozono S (2021). SARS-CoV-2 D614G spike mutation increases entry efficiency with enhanced ACE2-binding affinity. Nat Commun.

[42] Wu L (2022). SARS-CoV-2 Omicron RBD shows weaker binding affinity than the currently dominant Delta variant to human ACE2. Signal Transduct Target Ther.

[43] Harvey WT (2021). SARS-CoV-2 variants, spike mutations and immune escape. Nature Reviews Microbiol.

[44] Davis C (2021). Reduced neutralisation of the Delta (B. 1.617. 2) SARS-CoV-2 variant of concern following vaccination. PLoS Pathog.

[45] Wall EC (2021). AZD1222-induced neutralising antibody activity against SARS-CoV-2 Delta VOC. Lancet.

[46] Garcia-Beltran WF (2021). Multiple SARS-CoV-2 variants escape neutralization by vaccine-induced humoral immunity. Cell.

[47] Bartsch YC (2023). Selective SARS-CoV2 BA. 2 escape of antibody Fc/Fc-receptor interactions. Iscience.

[48] Bartsch YC (2022). Omicron variant Spike-specific antibody binding and Fc activity are preserved in recipients of mRNA or inactivated COVID-19 vaccines. Sci Trans Med.

[49] Cao Y (2020). Comparative genetic analysis of the novel coronavirus (2019-nCoV/SARS-CoV-2) receptor ACE2 in different populations. Cell Discov.

[50] Ali F (2020). ACE2 coding variants in different populations and their potential impact on SARS-CoV-2 binding affinity. Biochem Biophys Rep.

[51] Chen F (2021). The impact of ACE2 polymorphisms on COVID-19 disease: susceptibility, severity, and therapy. Front Cell Infect Microbiol.

[52] Motozono C (2021). SARS-CoV-2 spike L452R variant evades cellular immunity and increases infectivity. Cell Host Microbe.

[53] Kutter JS (2021). SARS-CoV and SARS-CoV-2 are transmitted through the air between ferrets over more than one meter distance. Nat Commun.

[54] Richard M (2020). SARS-CoV-2 is transmitted via contact and via the air between ferrets. Nat Commun.

[55] Irrgang P (2022). Class switch towards non-inflammatory, spike-specific IgG4 antibodies after repeated SARS-CoV-2 mRNA vaccination. Sci Immunol.

[56] Koutsakos M (2022). The magnitude and timing of recalled immunity after breakthrough infection is shaped by SARS-CoV-2 variants. Immunity.

[57] Van Erp EA (2019). Fc-mediated antibody effector functions during respiratory syncytial virus infection and disease. Front Immunol.

[58] Parsons MS, Chung AW, Kent SJ (2018). Importance of Fc-mediated functions of anti-HIV-1 broadly neutralizing antibodies. Retrovirology.

[59] Spencer DA (2022). Phagocytosis by an HIV antibody is associated with reduced viremia irrespective of enhanced complement lysis. Nat Commun.

[60] Gunn BM (2021). A Fc engineering approach to define functional humoral correlates of immunity against Ebola virus. Immunity.

[61] Gartlan C (2022). Vaccine-associated enhanced disease and pathogenic human coronaviruses. Front Immunol.

