# Supplementary material for: Antibody Fc-binding profiles and ACE2 affinity to SARS-CoV-2 RBD variants
Source: Med Microbiol Immunol. 2023 Jul 21;212(4):291–305. doi: 10.1007/s00430-023-00773-w (PMC10372118; doi:10.1007/s00430-023-00773-w)
Supplement: Supplementary file 1 — Supplementary file1 Supplementary Figure 1. Schematic representation of duplex phagocytosis assay with gating strategy and reciprocal ED50 analysis. (A) Schematic of the antibody dependent phagocytosis (ADCP) duplex assay. Briefly, APC fluorescent beads coated with ancestral RBD, while FITC fluorescent beads coated with RBD Beta were added to wells and incubated with plasma for 2 hours at 37OC. Following incubation, THP-1 monocytes were added to wells and incubated with opsonised beads for 2 hours under cell culture conditions before fixing cells for flow cytometry. (B) Representative gating strategy using pooled 2-week post vaccination plasma (blue), pooled baseline plasma (red) and no plasma (grey). Gating was performed by gating on THP-1 monocytes, single cells and finally cells positive for RBDWT beads and RBD Beta beads. (C) Visualization of single and double bead positive cells as dot plots for representative samples. ADCP by THP-1 monocytes induced by the plasma of BNT162b2 (two-weeks following second dose; n = 16) against the ancestral and Beta RBD shown as phagocytic score reciprocal ED50. Reciprocal ED50 values of ADCP duplex assay (D) without baseline correction using no plasma control (no baseline correction) and (E) with baseline correction using matched plasma samples collected prior to vaccination (baseline correction). (F) Pearson correlation of ED50 values calculated with no baseline correction and baseline correction. Supplementary Figure 2. SARS-CoV-2 serological signatures distinguish BNT162b2 vaccinated, convalescent, and baseline individuals. Plasma humoral responses were profiled via multiplex for convalescent mild/moderate COVID-19 patients (green square; median 38 days post symptom onset; n = 15) and BNT162b2-vaccinated individuals at baseline (purple triangle; n = 16) and two-weeks following second dose (blue circle; n = 16). Radar plots show the median MFI (median fluorescence intensity) value of each antibody signature (IgG, IgA, IgM, IgG [file 430_2023_773_MOESM1_ESM.pptx]

## Slide 1
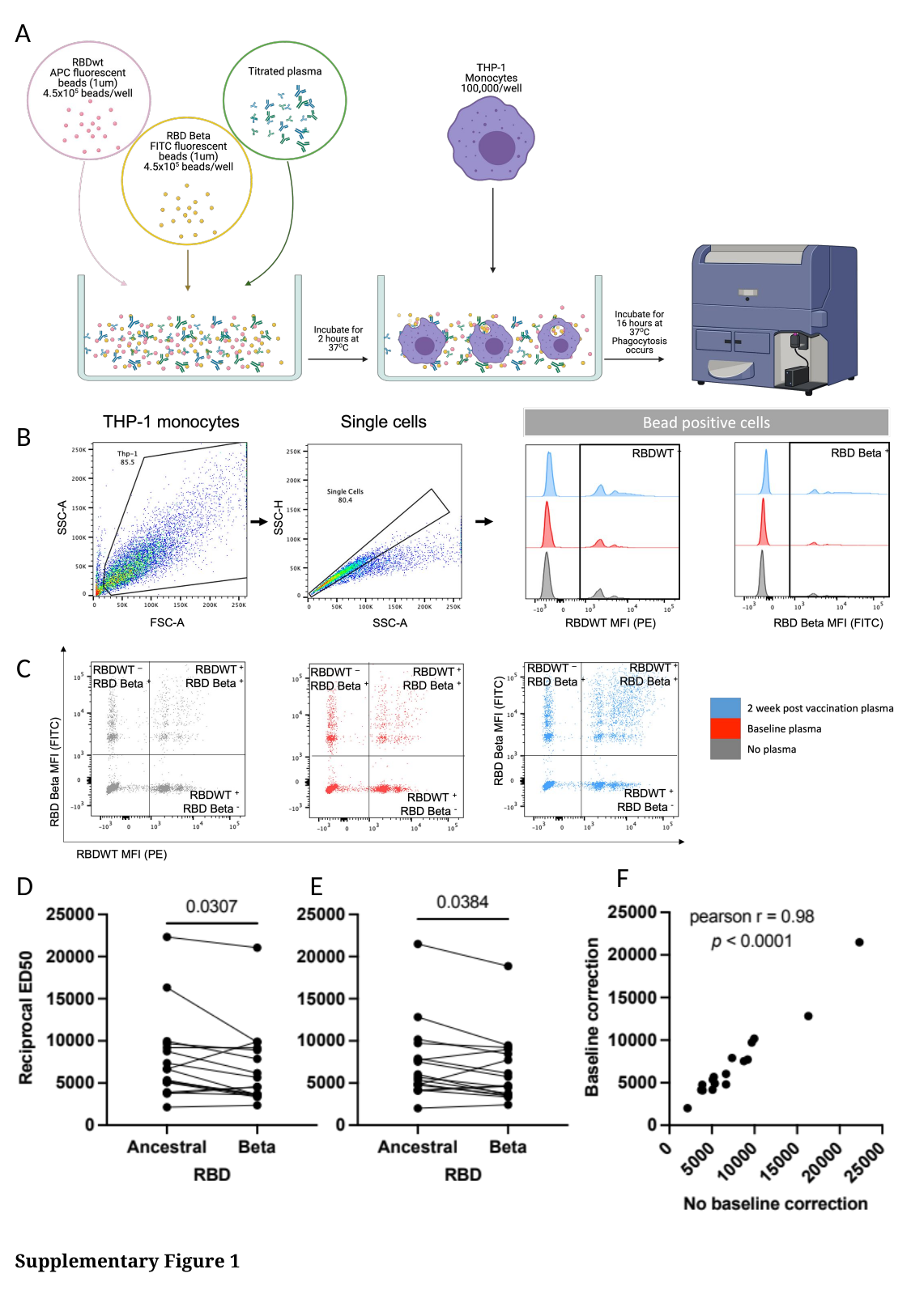

A
B
F
D
E
C
Supplementary Figure 1

## Slide 2
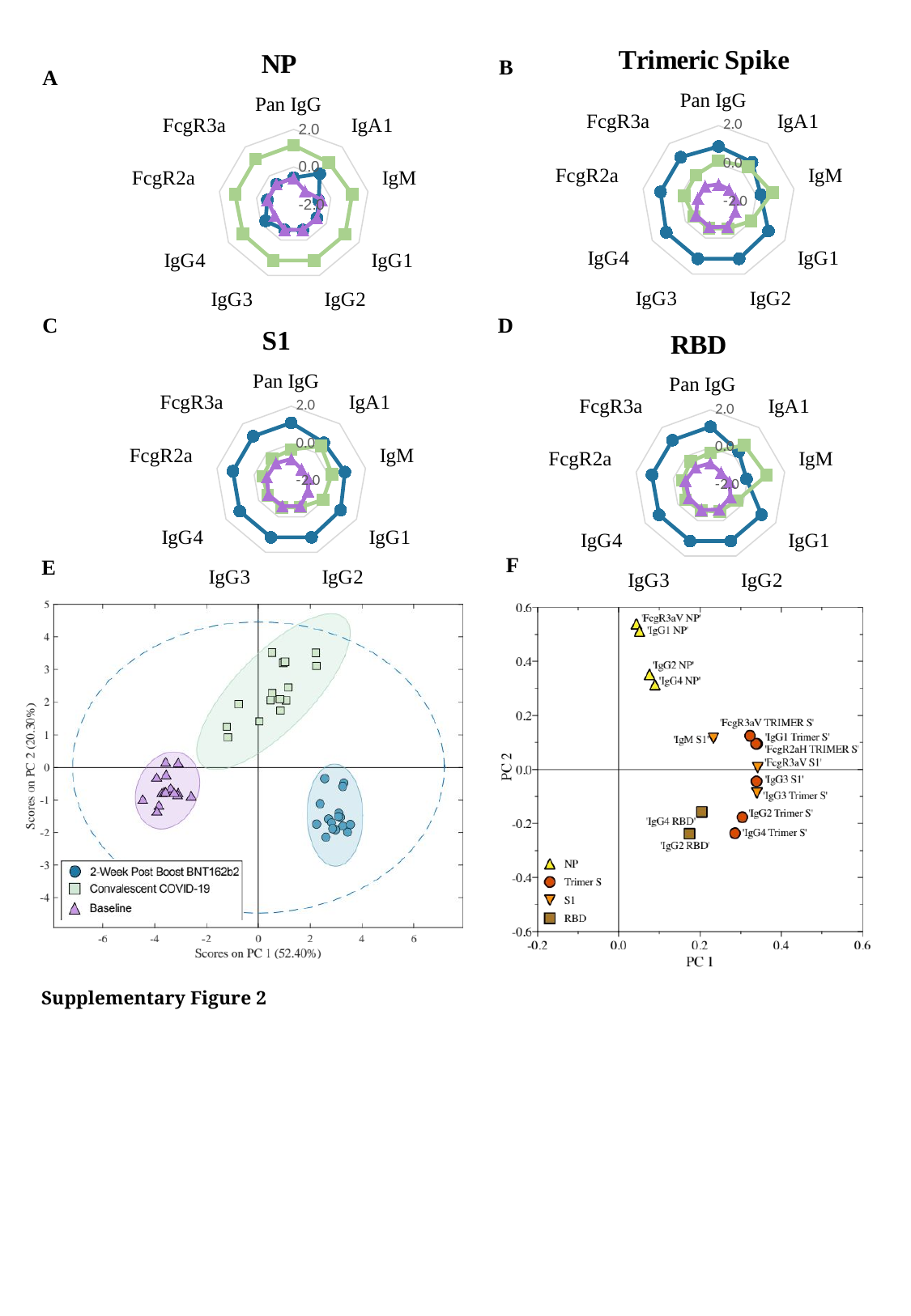

### Chart: Trimeric Spike
| Category | 2-Week Post Boost | Convalescent | Baseline |
|---|---|---|---|
| Pan IgG | 0.907764091555055 | 0.164157811806447 | -1.0719219033615 |
| IgA1 | 0.727201121193963 | 0.413177333656378 | -1.14037845485034 |
| IgM | 0.238154293310688 | 0.859422674209875 | -1.09757696752056 |
| IgG1 | 1.01409297517113 | -0.0288084942376742 | -0.985284480933459 |
| IgG2 | 1.15350453505142 | -0.531249897127526 | -0.622254637923896 |
| IgG3 | 1.15390613046613 | -0.539865580086185 | -0.614040550379941 |
| IgG4 | 1.15227716950215 | -0.511385256781936 | -0.640891912720218 |
| FcgR2a | 1.07600257111191 | -0.175146148868565 | -0.900856422243343 |
| FcgR3a | 1.06953758632595 | -0.157851012238493 | -0.911686574087462 |
### Chart: NP
| Category | 2-Week Post Boost | Convalescent | Baseline |
|---|---|---|---|
| Pan IgG | -0.566729242118037 | 1.15463580421397 | -0.587906562095937 |
| IgA1 | 0.153361708279312 | 0.91445997330377 | -1.06782168158308 |
| IgM | -0.649532290047012 | 1.1515556304064 | -0.50202334035939 |
| IgG1 | -0.579348316619815 | 1.15469823082023 | -0.575349914200413 |
| IgG2 | -0.571346752758778 | 1.15467980102998 | -0.5833330482712 |
| IgG3 | -0.575079528111243 | 1.15469756530088 | -0.579618037189638 |
| IgG4 | -0.277350098112615 | 1.10940039245046 | -0.832050294337844 |
| FcgR2a | -0.575960739419875 | 1.15469942452827 | -0.578738685108395 |
| FcgR3a | -0.5805157410183 | 1.15469474259553 | -0.574179001577227 |B
A
D
C
### Chart: S1
| Category | 2-Week Post Boost | Convalescent | Baseline |
|---|---|---|---|
| Pan IgG | 1.11728519955731 | -0.30614440743337 | -0.81114079212394 |
| IgA1 | 0.692471449401949 | 0.453990786246004 | -1.14646223564795 |
| IgM | 0.890183963951205 | 0.191837670924222 | -1.08202163487543 |
| IgG1 | 1.02852108316476 | -0.0597186995984073 | -0.968802383566357 |
| IgG2 | 1.15419186385303 | -0.547416712570296 | -0.606775151282738 |
| IgG3 | 1.15411631751653 | -0.545251803551117 | -0.608864513965413 |
| IgG4 | 1.15438150320177 | -0.553685245879583 | -0.600696257322189 |
| FcgR2a | 1.14688825694468 | -0.45731706633004 | -0.689571190614636 |
| FcgR3a | 1.13995398951808 | -0.410670132583043 | -0.729283856935036 |
### Chart: RBD
| Category | 2-Week Post Boost | Convalescent | Baseline |
|---|---|---|---|
| Pan IgG | 1.10871237855634 | -0.274950308475771 | -0.833762070080566 |
| IgA1 | 0.329581188245216 | 0.79361025117447 | -1.12319143941969 |
| IgM | -0.054159855691273 | 1.02597933845895 | -0.971819482767677 |
| IgG1 | 1.13051912187942 | -0.361679389265912 | -0.768839732613511 |
| IgG2 | 1.15328921105814 | -0.527217925055149 | -0.62607128600299 |
| IgG3 | 1.15434722085197 | -0.552437598550586 | -0.601909622301385 |
| IgG4 | 1.14799360533468 | -0.466372402167214 | -0.681621203167467 |
| FcgR2a | 1.15107990673536 | -0.496411663656732 | -0.654668243078632 |
| FcgR3a | 1.1296482554698 | -0.357650012108622 | -0.771998243361177 |F
E
Supplementary Figure 2

## Slide 3
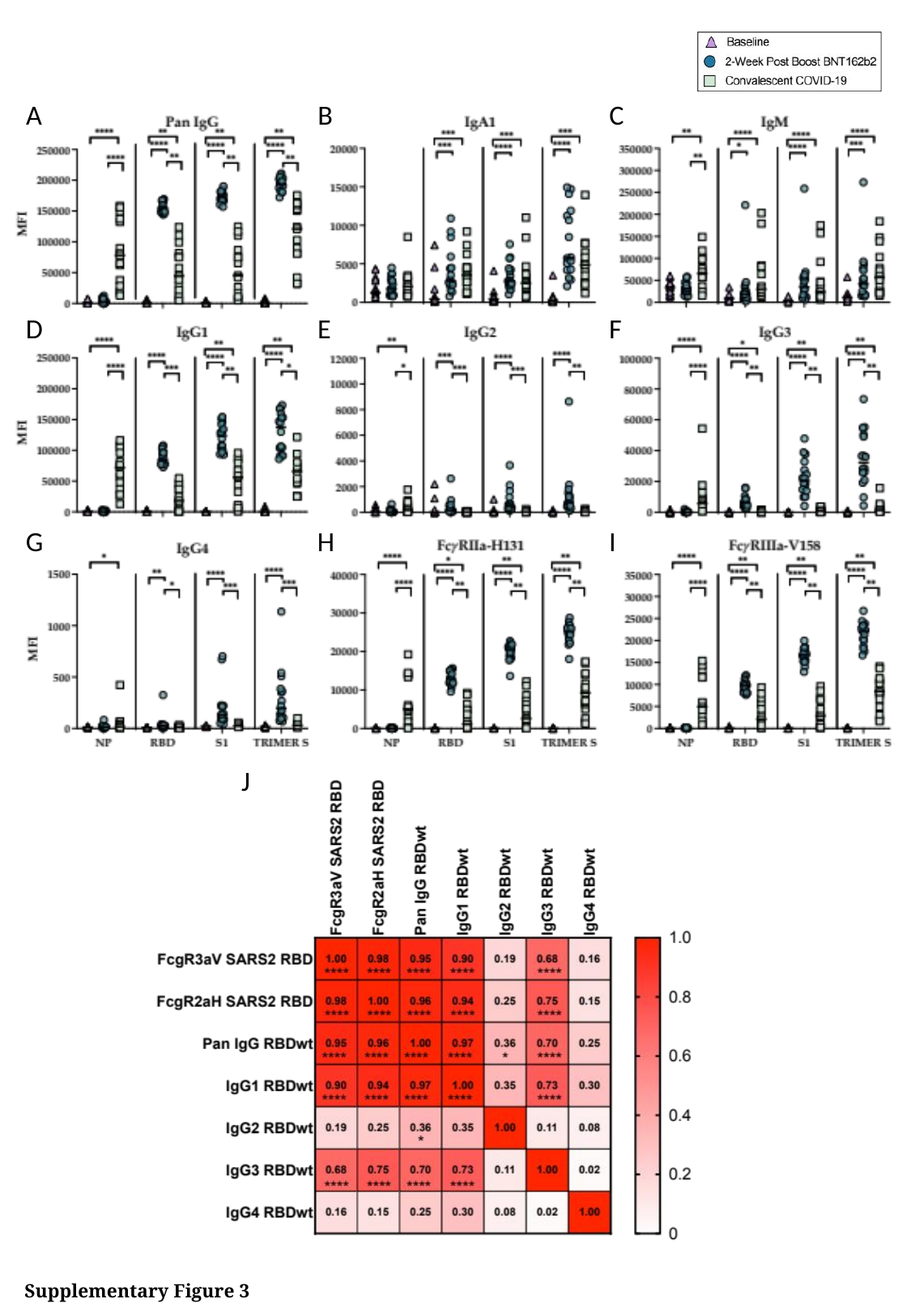

A
B
C
D
E
F
G
H
I
J
Supplementary Figure 3

## Slide 4
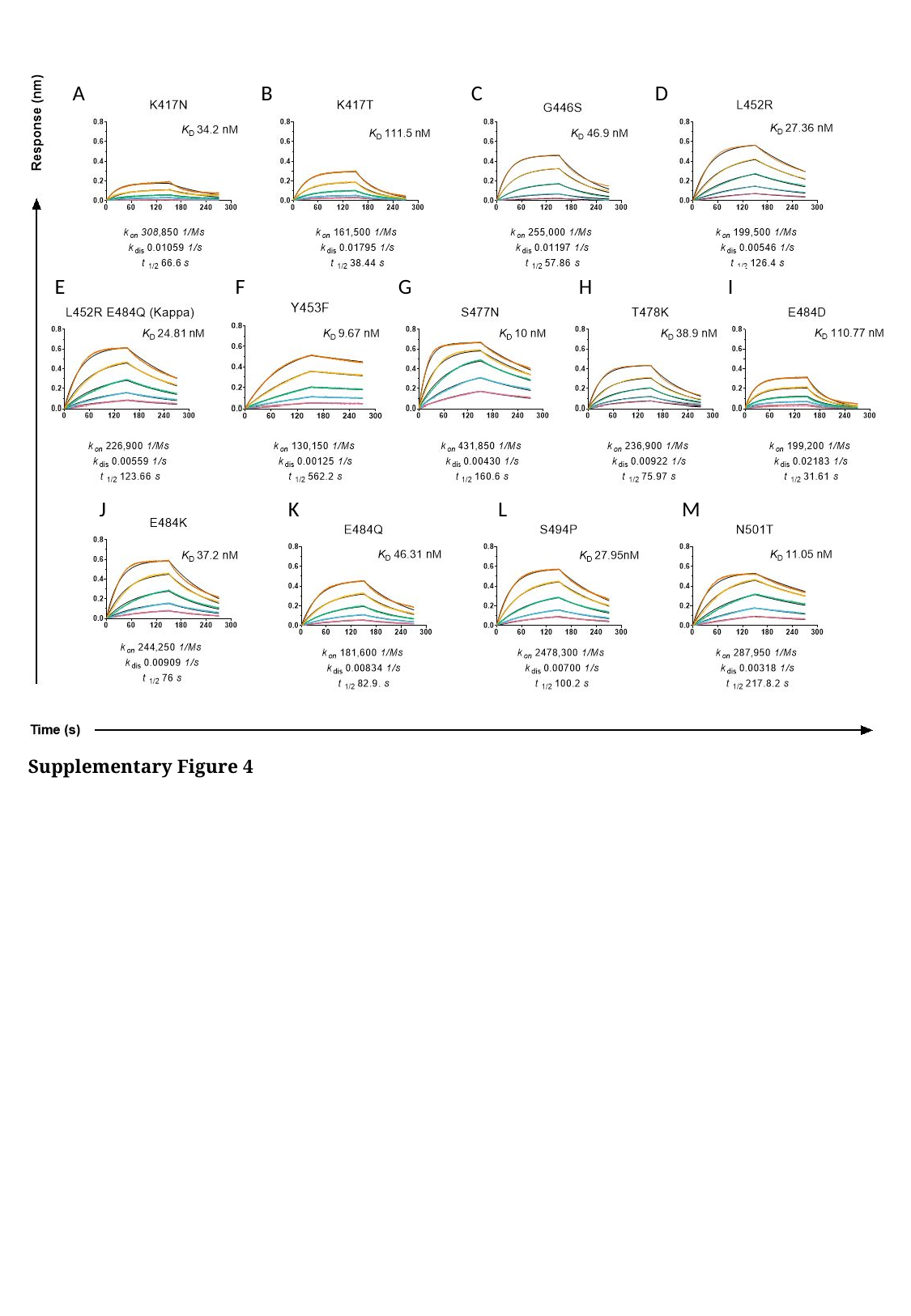

A
B
C
D
I
E
F
G
H
J
K
L
M
Supplementary Figure 4

## Slide 5
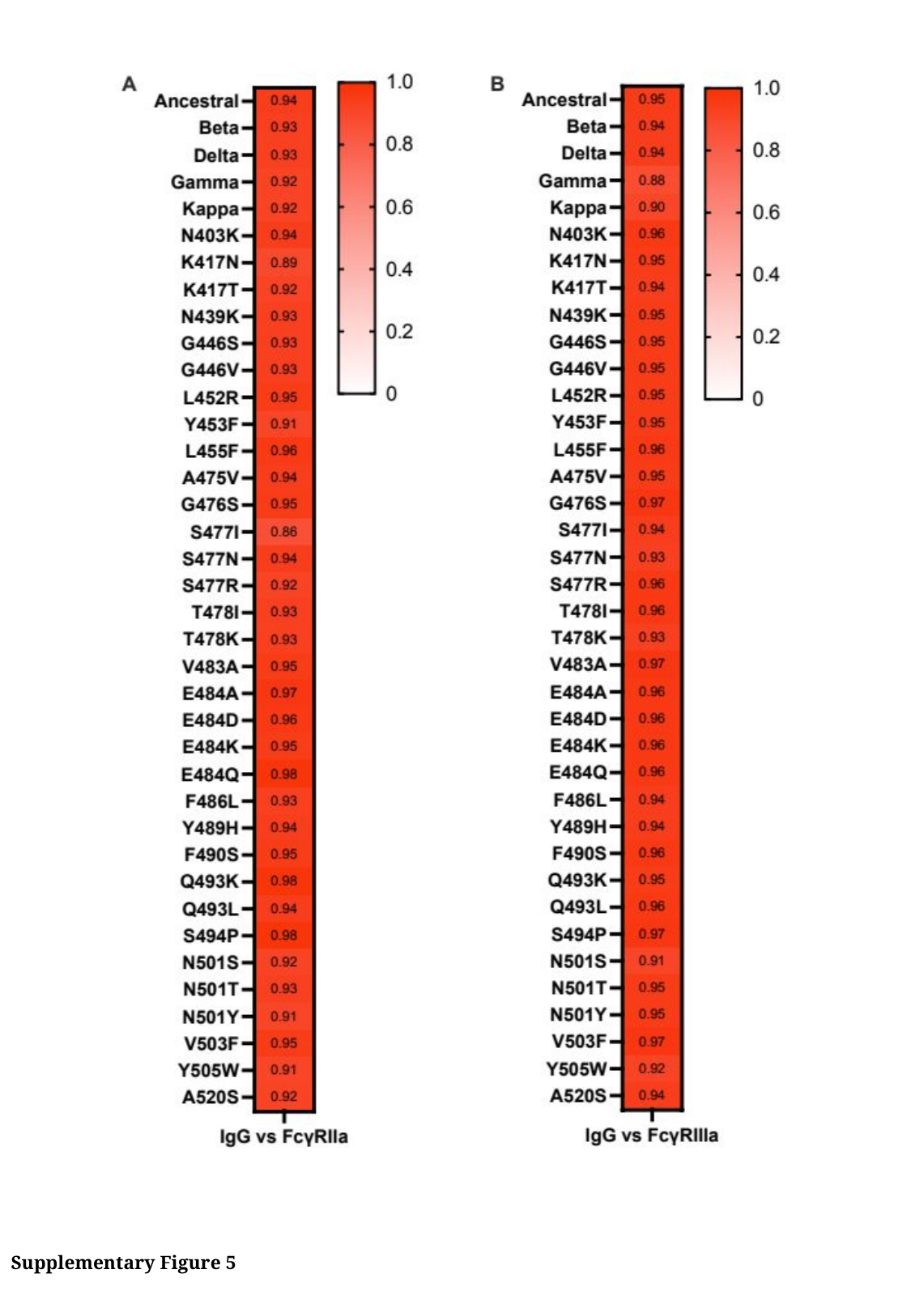

Supplementary Figure 5

## Slide 6
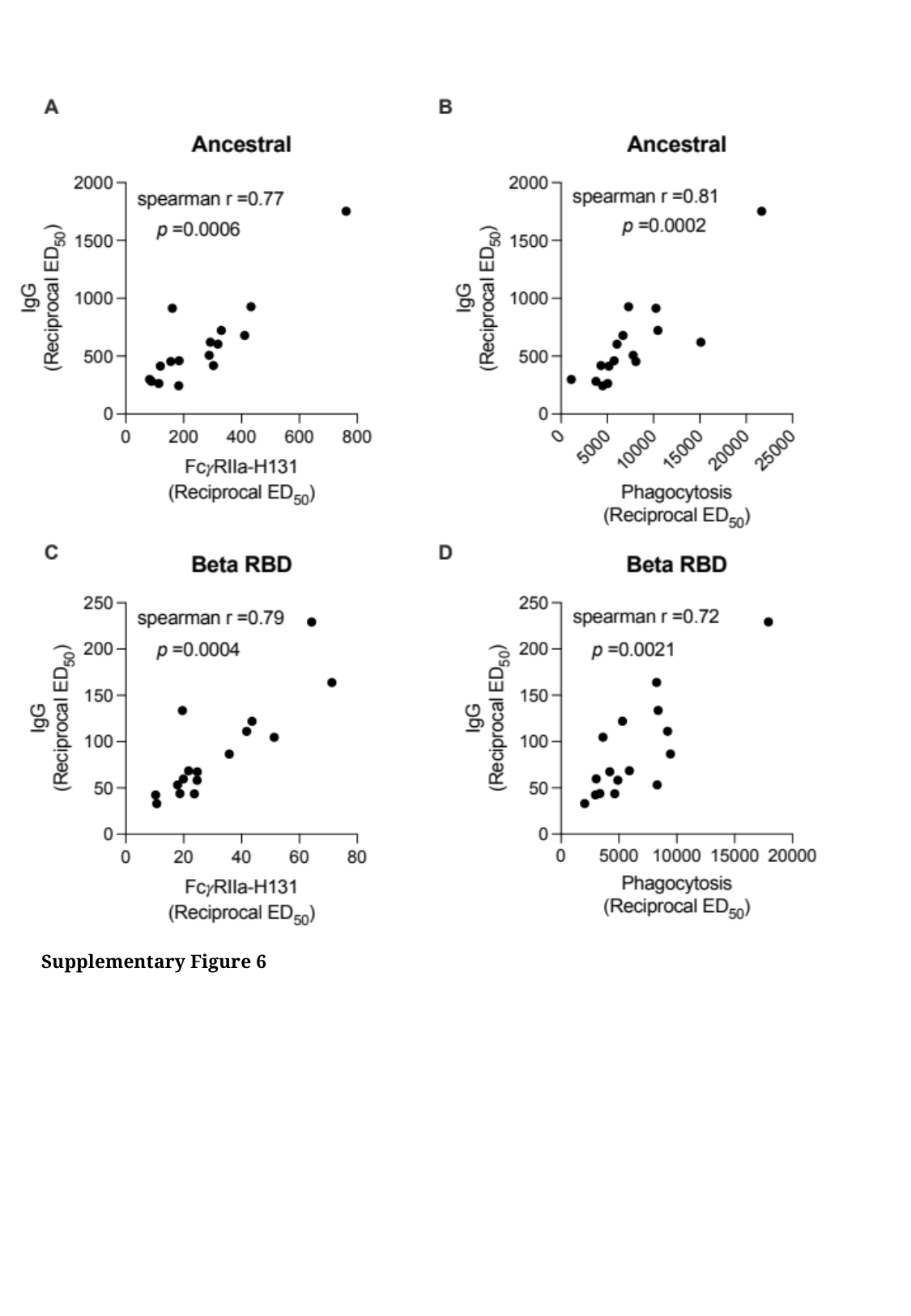

Supplementary Figure 6

## Slide 7
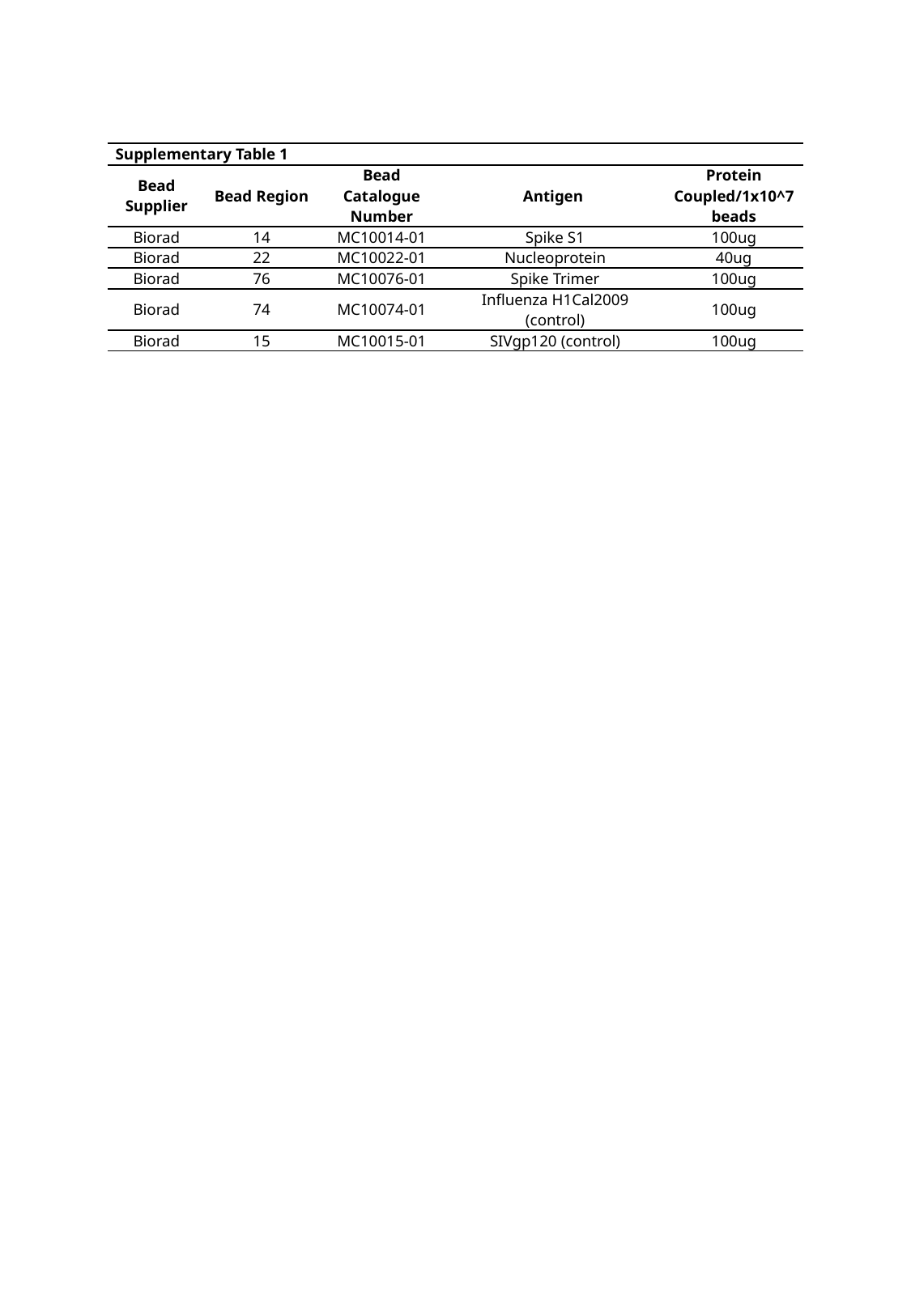

| Supplementary Table 1 | | | | |
| --- | --- | --- | --- | --- |
| Bead Supplier | Bead Region | Bead Catalogue Number | Antigen | Protein Coupled/1x10^7 beads |
| Biorad | 14 | MC10014-01 | Spike S1 | 100ug |
| Biorad | 22 | MC10022-01 | Nucleoprotein | 40ug |
| Biorad | 76 | MC10076-01 | Spike Trimer | 100ug |
| Biorad | 74 | MC10074-01 | Influenza H1Cal2009 (control) | 100ug |
| Biorad | 15 | MC10015-01 | SIVgp120 (control) | 100ug |

## Slide 8
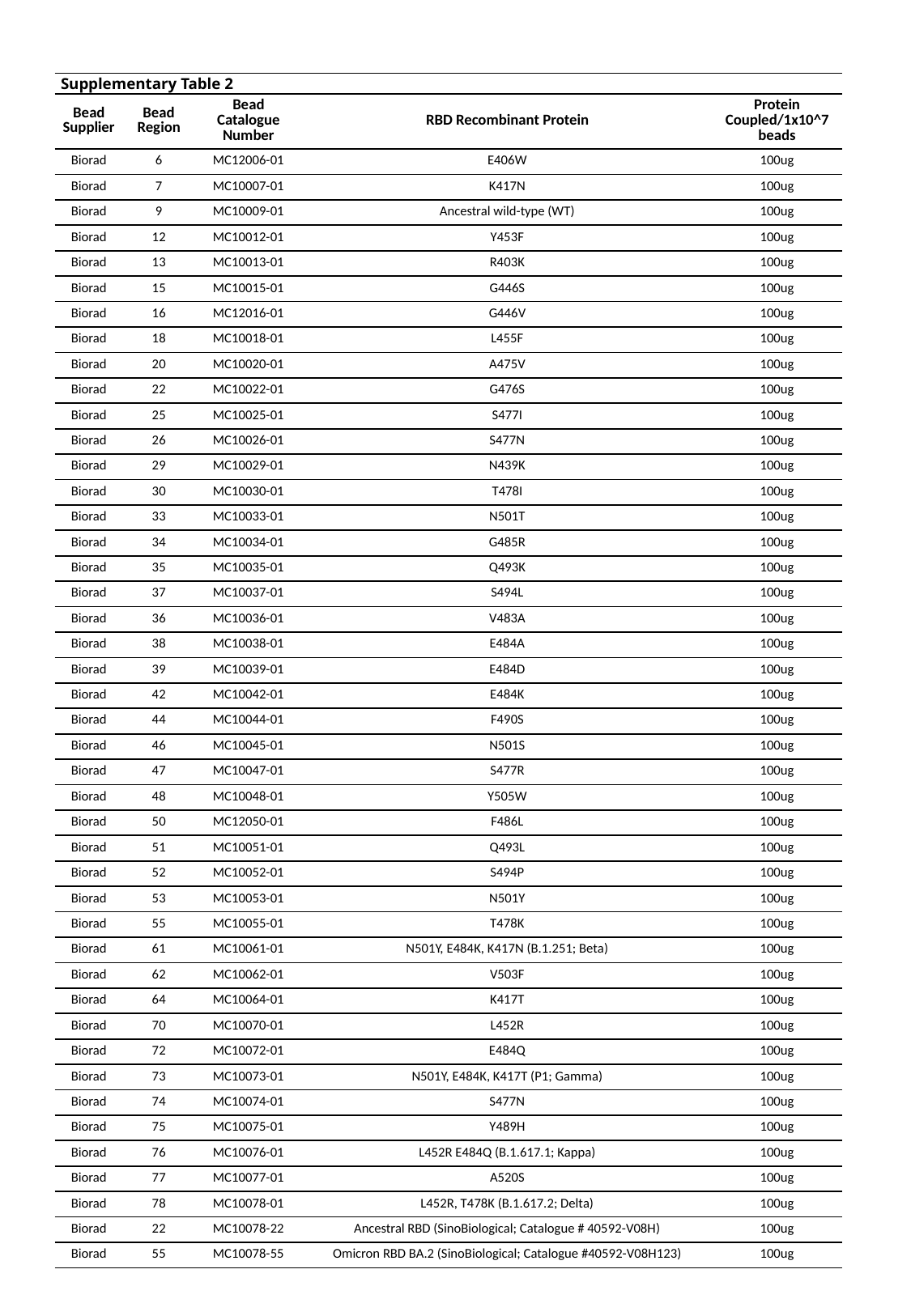

| Supplementary Table 2 | | | | |
| --- | --- | --- | --- | --- |
| Bead Supplier | Bead Region | Bead Catalogue Number | RBD Recombinant Protein | Protein Coupled/1x10^7 beads |
| Biorad | 6 | MC12006-01 | E406W | 100ug |
| Biorad | 7 | MC10007-01 | K417N | 100ug |
| Biorad | 9 | MC10009-01 | Ancestral wild-type (WT) | 100ug |
| Biorad | 12 | MC10012-01 | Y453F | 100ug |
| Biorad | 13 | MC10013-01 | R403K | 100ug |
| Biorad | 15 | MC10015-01 | G446S | 100ug |
| Biorad | 16 | MC12016-01 | G446V | 100ug |
| Biorad | 18 | MC10018-01 | L455F | 100ug |
| Biorad | 20 | MC10020-01 | A475V | 100ug |
| Biorad | 22 | MC10022-01 | G476S | 100ug |
| Biorad | 25 | MC10025-01 | S477I | 100ug |
| Biorad | 26 | MC10026-01 | S477N | 100ug |
| Biorad | 29 | MC10029-01 | N439K | 100ug |
| Biorad | 30 | MC10030-01 | T478I | 100ug |
| Biorad | 33 | MC10033-01 | N501T | 100ug |
| Biorad | 34 | MC10034-01 | G485R | 100ug |
| Biorad | 35 | MC10035-01 | Q493K | 100ug |
| Biorad | 37 | MC10037-01 | S494L | 100ug |
| Biorad | 36 | MC10036-01 | V483A | 100ug |
| Biorad | 38 | MC10038-01 | E484A | 100ug |
| Biorad | 39 | MC10039-01 | E484D | 100ug |
| Biorad | 42 | MC10042-01 | E484K | 100ug |
| Biorad | 44 | MC10044-01 | F490S | 100ug |
| Biorad | 46 | MC10045-01 | N501S | 100ug |
| Biorad | 47 | MC10047-01 | S477R | 100ug |
| Biorad | 48 | MC10048-01 | Y505W | 100ug |
| Biorad | 50 | MC12050-01 | F486L | 100ug |
| Biorad | 51 | MC10051-01 | Q493L | 100ug |
| Biorad | 52 | MC10052-01 | S494P | 100ug |
| Biorad | 53 | MC10053-01 | N501Y | 100ug |
| Biorad | 55 | MC10055-01 | T478K | 100ug |
| Biorad | 61 | MC10061-01 | N501Y, E484K, K417N (B.1.251; Beta) | 100ug |
| Biorad | 62 | MC10062-01 | V503F | 100ug |
| Biorad | 64 | MC10064-01 | K417T | 100ug |
| Biorad | 70 | MC10070-01 | L452R | 100ug |
| Biorad | 72 | MC10072-01 | E484Q | 100ug |
| Biorad | 73 | MC10073-01 | N501Y, E484K, K417T (P1; Gamma) | 100ug |
| Biorad | 74 | MC10074-01 | S477N | 100ug |
| Biorad | 75 | MC10075-01 | Y489H | 100ug |
| Biorad | 76 | MC10076-01 | L452R E484Q (B.1.617.1; Kappa) | 100ug |
| Biorad | 77 | MC10077-01 | A520S | 100ug |
| Biorad | 78 | MC10078-01 | L452R, T478K (B.1.617.2; Delta) | 100ug |
| Biorad | 22 | MC10078-22 | Ancestral RBD (SinoBiological; Catalogue # 40592-V08H) | 100ug |
| Biorad | 55 | MC10078-55 | Omicron RBD BA.2 (SinoBiological; Catalogue #40592-V08H123) | 100ug |

## Slide 9
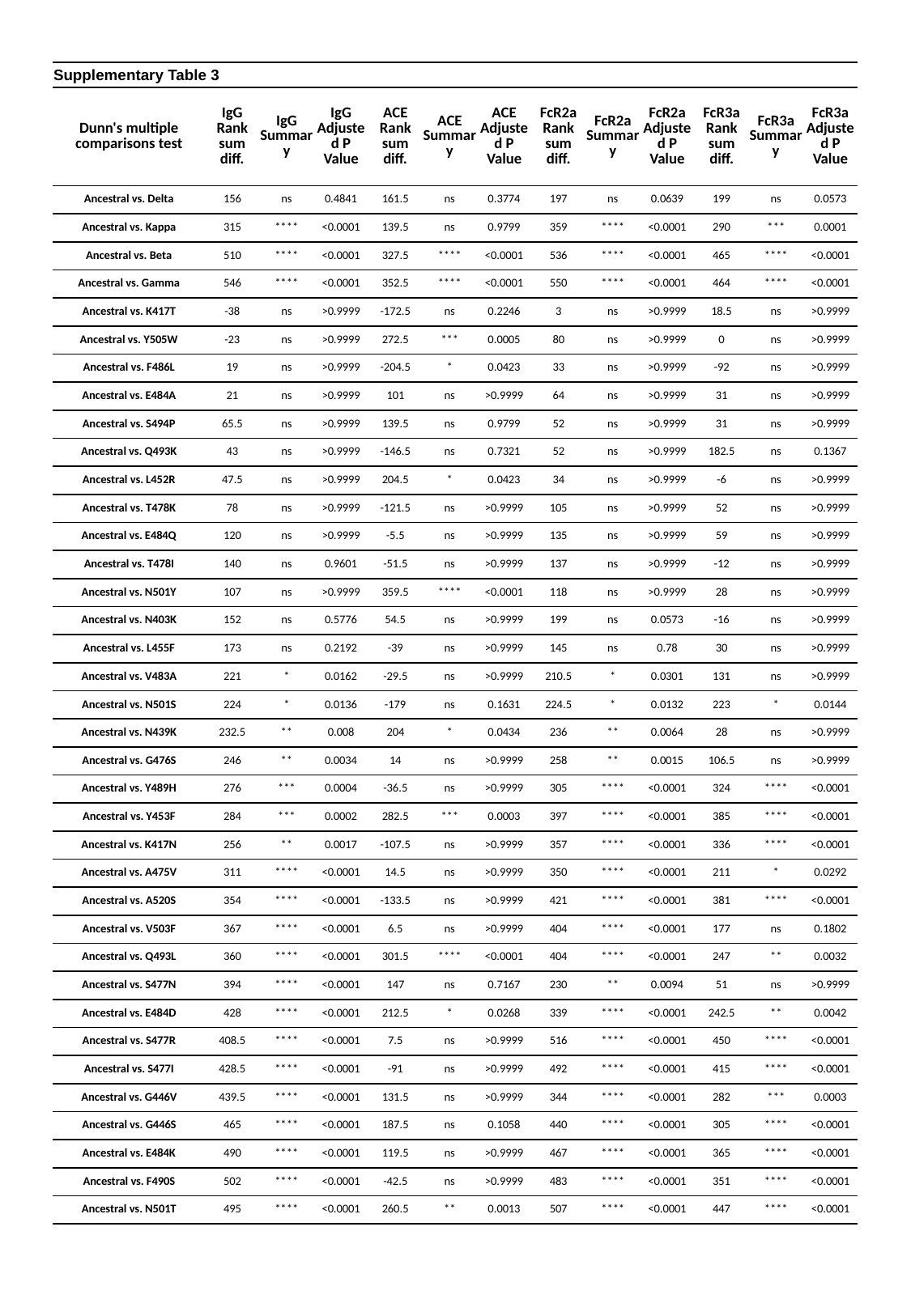

| Supplementary Table 3 | | | | | | | | | | | | |
| --- | --- | --- | --- | --- | --- | --- | --- | --- | --- | --- | --- | --- |
| Dunn's multiple comparisons test | IgG Rank sum diff. | IgG Summary | IgG Adjusted P Value | ACE Rank sum diff. | ACE Summary | ACE Adjusted P Value | FcR2a Rank sum diff. | FcR2a Summary | FcR2a Adjusted P Value | FcR3a Rank sum diff. | FcR3a Summary | FcR3a Adjusted P Value |
| Ancestral vs. Delta | 156 | ns | 0.4841 | 161.5 | ns | 0.3774 | 197 | ns | 0.0639 | 199 | ns | 0.0573 |
| Ancestral vs. Kappa | 315 | \*\*\*\* | <0.0001 | 139.5 | ns | 0.9799 | 359 | \*\*\*\* | <0.0001 | 290 | \*\*\* | 0.0001 |
| Ancestral vs. Beta | 510 | \*\*\*\* | <0.0001 | 327.5 | \*\*\*\* | <0.0001 | 536 | \*\*\*\* | <0.0001 | 465 | \*\*\*\* | <0.0001 |
| Ancestral vs. Gamma | 546 | \*\*\*\* | <0.0001 | 352.5 | \*\*\*\* | <0.0001 | 550 | \*\*\*\* | <0.0001 | 464 | \*\*\*\* | <0.0001 |
| Ancestral vs. K417T | -38 | ns | >0.9999 | -172.5 | ns | 0.2246 | 3 | ns | >0.9999 | 18.5 | ns | >0.9999 |
| Ancestral vs. Y505W | -23 | ns | >0.9999 | 272.5 | \*\*\* | 0.0005 | 80 | ns | >0.9999 | 0 | ns | >0.9999 |
| Ancestral vs. F486L | 19 | ns | >0.9999 | -204.5 | \* | 0.0423 | 33 | ns | >0.9999 | -92 | ns | >0.9999 |
| Ancestral vs. E484A | 21 | ns | >0.9999 | 101 | ns | >0.9999 | 64 | ns | >0.9999 | 31 | ns | >0.9999 |
| Ancestral vs. S494P | 65.5 | ns | >0.9999 | 139.5 | ns | 0.9799 | 52 | ns | >0.9999 | 31 | ns | >0.9999 |
| Ancestral vs. Q493K | 43 | ns | >0.9999 | -146.5 | ns | 0.7321 | 52 | ns | >0.9999 | 182.5 | ns | 0.1367 |
| Ancestral vs. L452R | 47.5 | ns | >0.9999 | 204.5 | \* | 0.0423 | 34 | ns | >0.9999 | -6 | ns | >0.9999 |
| Ancestral vs. T478K | 78 | ns | >0.9999 | -121.5 | ns | >0.9999 | 105 | ns | >0.9999 | 52 | ns | >0.9999 |
| Ancestral vs. E484Q | 120 | ns | >0.9999 | -5.5 | ns | >0.9999 | 135 | ns | >0.9999 | 59 | ns | >0.9999 |
| Ancestral vs. T478I | 140 | ns | 0.9601 | -51.5 | ns | >0.9999 | 137 | ns | >0.9999 | -12 | ns | >0.9999 |
| Ancestral vs. N501Y | 107 | ns | >0.9999 | 359.5 | \*\*\*\* | <0.0001 | 118 | ns | >0.9999 | 28 | ns | >0.9999 |
| Ancestral vs. N403K | 152 | ns | 0.5776 | 54.5 | ns | >0.9999 | 199 | ns | 0.0573 | -16 | ns | >0.9999 |
| Ancestral vs. L455F | 173 | ns | 0.2192 | -39 | ns | >0.9999 | 145 | ns | 0.78 | 30 | ns | >0.9999 |
| Ancestral vs. V483A | 221 | \* | 0.0162 | -29.5 | ns | >0.9999 | 210.5 | \* | 0.0301 | 131 | ns | >0.9999 |
| Ancestral vs. N501S | 224 | \* | 0.0136 | -179 | ns | 0.1631 | 224.5 | \* | 0.0132 | 223 | \* | 0.0144 |
| Ancestral vs. N439K | 232.5 | \*\* | 0.008 | 204 | \* | 0.0434 | 236 | \*\* | 0.0064 | 28 | ns | >0.9999 |
| Ancestral vs. G476S | 246 | \*\* | 0.0034 | 14 | ns | >0.9999 | 258 | \*\* | 0.0015 | 106.5 | ns | >0.9999 |
| Ancestral vs. Y489H | 276 | \*\*\* | 0.0004 | -36.5 | ns | >0.9999 | 305 | \*\*\*\* | <0.0001 | 324 | \*\*\*\* | <0.0001 |
| Ancestral vs. Y453F | 284 | \*\*\* | 0.0002 | 282.5 | \*\*\* | 0.0003 | 397 | \*\*\*\* | <0.0001 | 385 | \*\*\*\* | <0.0001 |
| Ancestral vs. K417N | 256 | \*\* | 0.0017 | -107.5 | ns | >0.9999 | 357 | \*\*\*\* | <0.0001 | 336 | \*\*\*\* | <0.0001 |
| Ancestral vs. A475V | 311 | \*\*\*\* | <0.0001 | 14.5 | ns | >0.9999 | 350 | \*\*\*\* | <0.0001 | 211 | \* | 0.0292 |
| Ancestral vs. A520S | 354 | \*\*\*\* | <0.0001 | -133.5 | ns | >0.9999 | 421 | \*\*\*\* | <0.0001 | 381 | \*\*\*\* | <0.0001 |
| Ancestral vs. V503F | 367 | \*\*\*\* | <0.0001 | 6.5 | ns | >0.9999 | 404 | \*\*\*\* | <0.0001 | 177 | ns | 0.1802 |
| Ancestral vs. Q493L | 360 | \*\*\*\* | <0.0001 | 301.5 | \*\*\*\* | <0.0001 | 404 | \*\*\*\* | <0.0001 | 247 | \*\* | 0.0032 |
| Ancestral vs. S477N | 394 | \*\*\*\* | <0.0001 | 147 | ns | 0.7167 | 230 | \*\* | 0.0094 | 51 | ns | >0.9999 |
| Ancestral vs. E484D | 428 | \*\*\*\* | <0.0001 | 212.5 | \* | 0.0268 | 339 | \*\*\*\* | <0.0001 | 242.5 | \*\* | 0.0042 |
| Ancestral vs. S477R | 408.5 | \*\*\*\* | <0.0001 | 7.5 | ns | >0.9999 | 516 | \*\*\*\* | <0.0001 | 450 | \*\*\*\* | <0.0001 |
| Ancestral vs. S477I | 428.5 | \*\*\*\* | <0.0001 | -91 | ns | >0.9999 | 492 | \*\*\*\* | <0.0001 | 415 | \*\*\*\* | <0.0001 |
| Ancestral vs. G446V | 439.5 | \*\*\*\* | <0.0001 | 131.5 | ns | >0.9999 | 344 | \*\*\*\* | <0.0001 | 282 | \*\*\* | 0.0003 |
| Ancestral vs. G446S | 465 | \*\*\*\* | <0.0001 | 187.5 | ns | 0.1058 | 440 | \*\*\*\* | <0.0001 | 305 | \*\*\*\* | <0.0001 |
| Ancestral vs. E484K | 490 | \*\*\*\* | <0.0001 | 119.5 | ns | >0.9999 | 467 | \*\*\*\* | <0.0001 | 365 | \*\*\*\* | <0.0001 |
| Ancestral vs. F490S | 502 | \*\*\*\* | <0.0001 | -42.5 | ns | >0.9999 | 483 | \*\*\*\* | <0.0001 | 351 | \*\*\*\* | <0.0001 |
| Ancestral vs. N501T | 495 | \*\*\*\* | <0.0001 | 260.5 | \*\* | 0.0013 | 507 | \*\*\*\* | <0.0001 | 447 | \*\*\*\* | <0.0001 |

## Slide 10
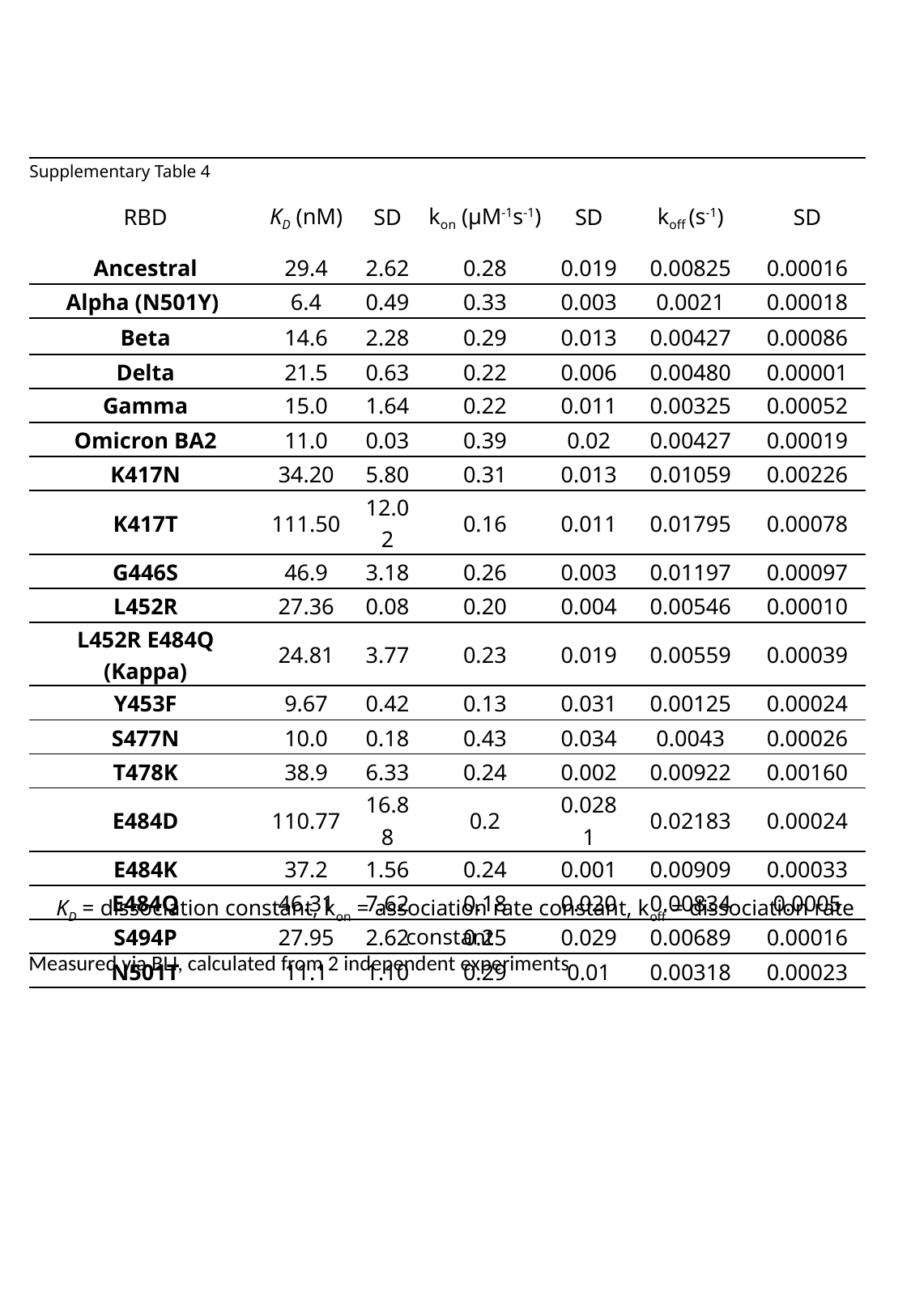

| Supplementary Table 4 | | | | | | |
| --- | --- | --- | --- | --- | --- | --- |
| RBD | KD (nM) | SD | kon (µM-1s-1) | SD | koff (s-1) | SD |
| Ancestral | 29.4 | 2.62 | 0.28 | 0.019 | 0.00825 | 0.00016 |
| Alpha (N501Y) | 6.4 | 0.49 | 0.33 | 0.003 | 0.0021 | 0.00018 |
| Beta | 14.6 | 2.28 | 0.29 | 0.013 | 0.00427 | 0.00086 |
| Delta | 21.5 | 0.63 | 0.22 | 0.006 | 0.00480 | 0.00001 |
| Gamma | 15.0 | 1.64 | 0.22 | 0.011 | 0.00325 | 0.00052 |
| Omicron BA2 | 11.0 | 0.03 | 0.39 | 0.02 | 0.00427 | 0.00019 |
| K417N | 34.20 | 5.80 | 0.31 | 0.013 | 0.01059 | 0.00226 |
| K417T | 111.50 | 12.02 | 0.16 | 0.011 | 0.01795 | 0.00078 |
| G446S | 46.9 | 3.18 | 0.26 | 0.003 | 0.01197 | 0.00097 |
| L452R | 27.36 | 0.08 | 0.20 | 0.004 | 0.00546 | 0.00010 |
| L452R E484Q (Kappa) | 24.81 | 3.77 | 0.23 | 0.019 | 0.00559 | 0.00039 |
| Y453F | 9.67 | 0.42 | 0.13 | 0.031 | 0.00125 | 0.00024 |
| S477N | 10.0 | 0.18 | 0.43 | 0.034 | 0.0043 | 0.00026 |
| T478K | 38.9 | 6.33 | 0.24 | 0.002 | 0.00922 | 0.00160 |
| E484D | 110.77 | 16.88 | 0.2 | 0.0281 | 0.02183 | 0.00024 |
| E484K | 37.2 | 1.56 | 0.24 | 0.001 | 0.00909 | 0.00033 |
| E484Q | 46.31 | 7.62 | 0.18 | 0.020 | 0.00834 | 0.0005 |
| S494P | 27.95 | 2.62 | 0.25 | 0.029 | 0.00689 | 0.00016 |
| N501T | 11.1 | 1.10 | 0.29 | 0.01 | 0.00318 | 0.00023 |
KD = dissociation constant, kon = association rate constant, koff = dissociation rate constant
Measured via BLI, calculated from 2 independent experiments

## Slide 11
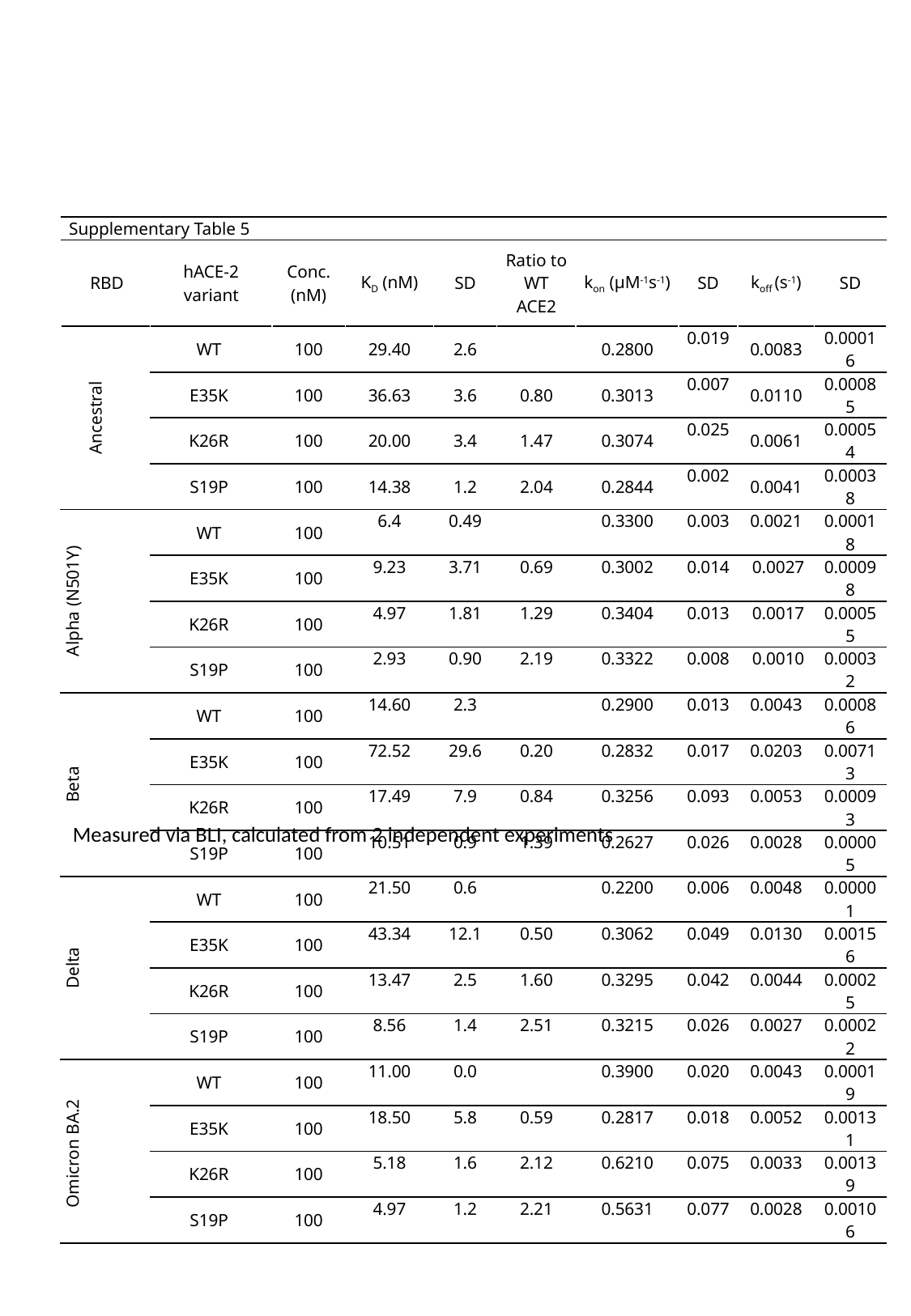

| Supplementary Table 5 | | | | | | | | | |
| --- | --- | --- | --- | --- | --- | --- | --- | --- | --- |
| RBD | hACE-2 variant | Conc. (nM) | KD (nM) | SD | Ratio to WT ACE2 | kon (µM-1s-1) | SD | koff (s-1) | SD |
| Ancestral | WT | 100 | 29.40 | 2.6 | | 0.2800 | 0.019 | 0.0083 | 0.00016 |
| | E35K | 100 | 36.63 | 3.6 | 0.80 | 0.3013 | 0.007 | 0.0110 | 0.00085 |
| | K26R | 100 | 20.00 | 3.4 | 1.47 | 0.3074 | 0.025 | 0.0061 | 0.00054 |
| | S19P | 100 | 14.38 | 1.2 | 2.04 | 0.2844 | 0.002 | 0.0041 | 0.00038 |
| Alpha (N501Y) | WT | 100 | 6.4 | 0.49 | | 0.3300 | 0.003 | 0.0021 | 0.00018 |
| | E35K | 100 | 9.23 | 3.71 | 0.69 | 0.3002 | 0.014 | 0.0027 | 0.00098 |
| | K26R | 100 | 4.97 | 1.81 | 1.29 | 0.3404 | 0.013 | 0.0017 | 0.00055 |
| | S19P | 100 | 2.93 | 0.90 | 2.19 | 0.3322 | 0.008 | 0.0010 | 0.00032 |
| Beta | WT | 100 | 14.60 | 2.3 | | 0.2900 | 0.013 | 0.0043 | 0.00086 |
| | E35K | 100 | 72.52 | 29.6 | 0.20 | 0.2832 | 0.017 | 0.0203 | 0.00713 |
| | K26R | 100 | 17.49 | 7.9 | 0.84 | 0.3256 | 0.093 | 0.0053 | 0.00093 |
| | S19P | 100 | 10.51 | 0.9 | 1.39 | 0.2627 | 0.026 | 0.0028 | 0.00005 |
| Delta | WT | 100 | 21.50 | 0.6 | | 0.2200 | 0.006 | 0.0048 | 0.00001 |
| | E35K | 100 | 43.34 | 12.1 | 0.50 | 0.3062 | 0.049 | 0.0130 | 0.00156 |
| | K26R | 100 | 13.47 | 2.5 | 1.60 | 0.3295 | 0.042 | 0.0044 | 0.00025 |
| | S19P | 100 | 8.56 | 1.4 | 2.51 | 0.3215 | 0.026 | 0.0027 | 0.00022 |
| Omicron BA.2 | WT | 100 | 11.00 | 0.0 | | 0.3900 | 0.020 | 0.0043 | 0.00019 |
| | E35K | 100 | 18.50 | 5.8 | 0.59 | 0.2817 | 0.018 | 0.0052 | 0.00131 |
| | K26R | 100 | 5.18 | 1.6 | 2.12 | 0.6210 | 0.075 | 0.0033 | 0.00139 |
| | S19P | 100 | 4.97 | 1.2 | 2.21 | 0.5631 | 0.077 | 0.0028 | 0.00106 |
Measured via BLI, calculated from 2 independent experiments

## Slide 12
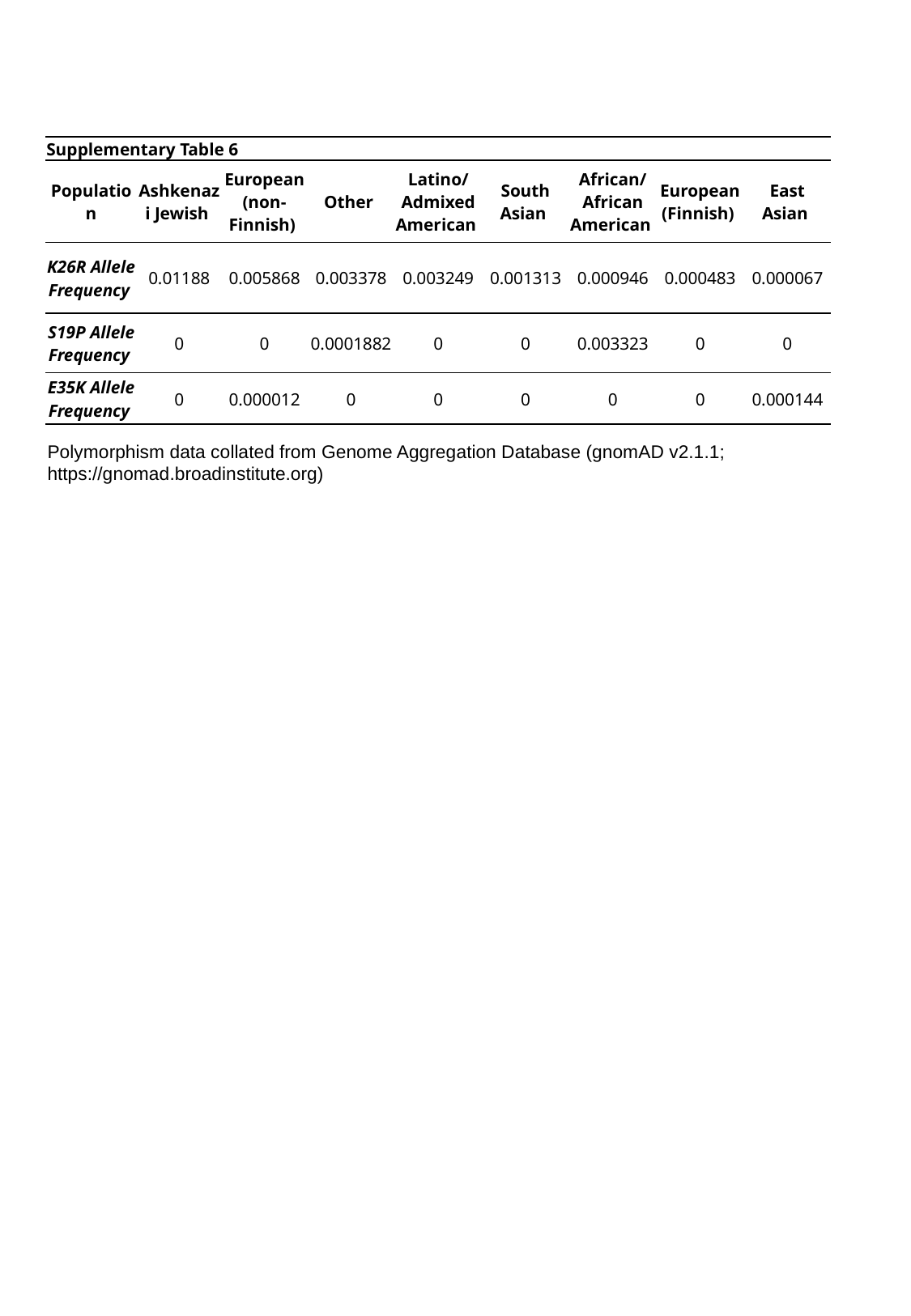

| Supplementary Table 6 | | | | | | | | |
| --- | --- | --- | --- | --- | --- | --- | --- | --- |
| Population | Ashkenazi Jewish | European (non-Finnish) | Other | Latino/Admixed American | South Asian | African/African American | European (Finnish) | East Asian |
| K26R Allele Frequency | 0.01188 | 0.005868 | 0.003378 | 0.003249 | 0.001313 | 0.000946 | 0.000483 | 0.000067 |
| S19P Allele Frequency | 0 | 0 | 0.0001882 | 0 | 0 | 0.003323 | 0 | 0 |
| E35K Allele Frequency | 0 | 0.000012 | 0 | 0 | 0 | 0 | 0 | 0.000144 |
Polymorphism data collated from Genome Aggregation Database (gnomAD v2.1.1; https://gnomad.broadinstitute.org)
